# Endothelial EGLN3-PKM2 signaling induces the formation of acute astrocytic barrier to alleviate immune cell infiltration after subarachnoid hemorrhage

**DOI:** 10.1186/s12987-024-00550-8

**Published:** 2024-05-16

**Authors:** Mingxu Duan, Xufang Ru, Jiru Zhou, Yuanshu Li, Peiwen Guo, Wenbo Kang, Wenyan Li, Zhi Chen, Hua Feng, Yujie Chen

**Affiliations:** 1 Department of Neurosurgery and State Key Laboratory of Trauma, Burn and Combined Injury, Southwest Hospital, Third Military Medical University (Army Medical University), 29 Gaotanyan Street, Shapingba District, Chongqing, 400038 China; 2 Chongqing Key Laboratory of Intelligent Diagnosis, Treatment and Rehabilitation of Central Nervous System Injuries, Southwest Hospital, Third Military Medical University (Army Medical University), Chongqing, 400038 China; 3 Chongqing Clinical Research Center for Neurosurgery, Southwest Hospital, Third Military Medical University (Army Medical University), Chongqing, 400038 China; 4 Department of Neurosurgery, The First Affiliated Hospital of Chongqing Medical University, Chongqing, 400016 China

**Keywords:** Subarachnoid hemorrhage, Glial limitans, Blood‒brain barrier, Immune cell infiltration, EGLN3

## Abstract

**Background:**

Most subarachnoid hemorrhage (SAH) patients have no obvious hematoma lesions but exhibit blood–brain barrier dysfunction and vasogenic brain edema. However, there is a few days between blood‒brain barrier dysfunction and vasogenic brain edema. The present study sought to investigate whether this phenomenon is caused by endothelial injury induced by the acute astrocytic barrier, also known as the glial limitans.

**Methods:**

Bioinformatics analyses of human endothelial cells and astrocytes under hypoxia were performed based on the GEO database. Wild-type, EGLN3 and PKM2 conditional knock-in mice were used to confirm glial limitan formation after SAH. Then, the effect of endothelial EGLN3-PKM2 signaling on temporal and spatial changes in glial limitans was evaluated in both in vivo and in vitro models of SAH.

**Results:**

The data indicate that in the acute phase after SAH, astrocytes can form a temporary protective barrier, the glia limitans, around blood vessels that helps maintain barrier function and improve neurological prognosis. Molecular docking studies have shown that endothelial cells and astrocytes can promote glial limitans-based protection against early brain injury through EGLN3/PKM2 signaling and further activation of the PKC/ERK/MAPK signaling pathway in astrocytes after SAH.

**Conclusion:**

Improving the ability to maintain glial limitans may be a new therapeutic strategy for improving the prognosis of SAH patients.

**Supplementary Information:**

The online version contains supplementary material available at 10.1186/s12987-024-00550-8.

## Background

Subarachnoid hemorrhage (SAH) is the third most common severe subtype of stroke, and aneurysms are the cause of SAH in 85% of cases [1]. Although existing treatments require a more accurate diagnosis, aneurysm surgery and the use of related medications have improved survival rates for subarachnoid hemorrhage by 17% over the past few decades [2]; however, there is no effective treatment to improve the prognosis of patients with secondary brain injury [3, 4]. Many studies have confirmed that blood‒brain barrier (BBB) disruption after SAH may be an important factor that leads to secondary brain edema and poor prognosis [5, 6]. Previous studies have demonstrated that although significant BBB damage can be observed as soon as 1 day after SAH [7], most brain edema in neurocritically ill patients occurs between 3 and 7 days after SAH, with a significant time difference between the two. This indicates that the mechanism and regularity of brain edema and neurological impairment after SAH need to be further studied.

An increasing number of studies have shown that astrocytes are important components of the BBB [8, 9]. Structurally, astrocytes are located outside microvessels, and their endfeet are wrapped around endothelial cells [10, 11]. Furthermore, in multiple sclerosis and experimental autoimmune encephalomyelitis models [12], astrocyte endfeet can form tight junctions similar to those of endothelial cells outside endothelial cells, called Glia limitans (GLs), which express CLDN1 in the endfeet, while CLDN4 and JAM-A [13] may limit the infiltration of inflammatory cells such as T lymphocytes into the brain parenchyma [14, 15]. Additional research has shown that the BBB is a complex barrier structure that includes GLs [12, 16]. This barrier structure regulates homeostasis of the central nervous system, maintains the dynamic balance of metabolism in the central nervous system, and protects the central nervous system from injury. However, determining the specific protective effect of GLs on the central nervous system and the factors influencing GL-mediated spatiotemporal changes after SAH still requires in-depth study.

The BBB is the most representative unit structure in the vascular neural network [17]. Early studies suggested that the BBB is mainly composed of cells, tight junctions between endothelial cells and the endothelial basement membrane [18, 19]. Martin, M. reported that the Wnt ligands Wnt7a and Wnt7b in endothelial cells not only play important roles in the development of the BBB but also play important roles in endothelial cell repair of the BBB after stroke [20]. More studies have shown that endothelial cells, as an important part of the vascular neural network, can maintain the homeostasis of the BBB through interactions with other cells in healthy and diseased states. Who et al. found that endothelial cells can perform neuro-glia-vessel communication through Dab1 signaling [21]; moreover, these researchers utilized a linked organ-on-a-chip model of the human neurovascular unit to discover metabolic coupling between endothelial cells and neurons [22]. Most importantly, after acute central system injury, endothelial cells interact with astrocytes, which play an important role in maintaining the homeostasis of the vascular neural network through paracrine, intracellular, and extracellular transport [23]. However, the effect of endothelial cells on the formation of astrocyte GLs after SAH and their protective effect on the central nervous system remain unknown.

EGLN3 is a proline hydroxylase that mediates the hydroxylation of proline residues in target proteins, such as PKM and HIF-1α [24–27], and can act as a cellular oxygen sensor in the HIF1-α signaling pathway to induce the cellular response to hypoxia [28, 29]. EGLN3 is expressed in a variety of cells, including neurons, endothelial cells [30], neutrophils and other cells, and is responsible for DNA damage after hypoxia [31], apoptosis [32] and inflammatory infiltration [33]. PKM2, an important substrate of EGLN3, is a pyruvate kinase. There is strong evidence that EGLN3 amplifies HIF signaling by hydroxylating PKM2 to promote the cellular stress response to hypoxia [34]. Further studies have shown that EGLN3 hydroxylation of PKM2 can enhance the direct interaction between PKM2 and the HIF-1α subunit, thereby promoting the transactivation of HIF-1 target genes [24, 34]. Although the role of EGLN3-PKM2 signaling in hypoxic stress has been studied, there is a serious lack of research on the protective effect of EGLN3-PKM2 signaling on the BBB and the formation of GLs after central nervous system injury.

To further understand the protective mechanism of the BBB after SAH, based on bioinformatics analysis of existing datasets, we hypothesized that after SAH, endothelial cells respond to the injury stimulus and activate the astrocyte stress response to injury through EGLN3-PKM2 signaling [35], which may upregulate the expression of CLDN1 through the PKC-ERK-MAPK pathway [36, 37], promote the formation of tight junctions between astrocyte endfeet, and lead to the formation of temporary protective barrier GLs, thus delaying the emergence of brain edema and neurological damage; these findings provide a new therapeutic target for treatment after SAH.

## Materials and methods

### Experimental design

**Fig. 1:**
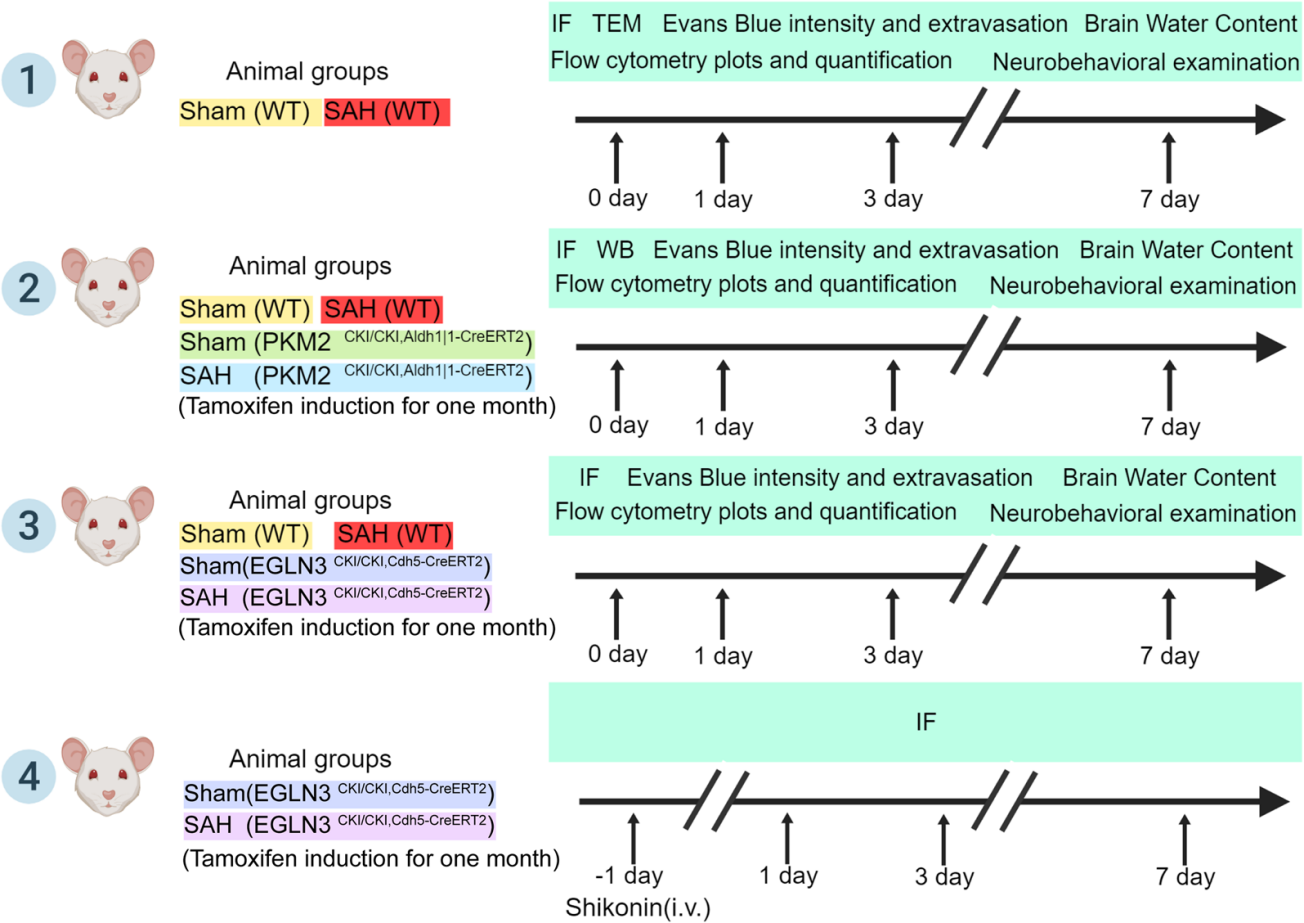
Schematic of the experimental design and subsequent analyses. IF indicates immunofluorescence staining; TEM indicates transmission electron microscopy; i.v. indicates intravenous injection; WB indicates Western blot; and EB indicates the Evans blue experiment

The experimental animals were divided into the following four groups: the WT subarachnoid hemorrhage group; the EGLN3^CKI/CKI, Cdh5−creERT2^ subarachnoid hemorrhage group; the PKM2^CKI/CKI, Aldh1|1−creERT2^ subarachnoid hemorrhage group; and the GLN 3^CKI/CKI, Cdh5−creERT2^ and injected with the PKM2 inhibitor Shikonin (Sigma, 817-89-5) after subarachnoid hemorrhage group (Fig. 1). The Shikonin dose and injection method were performed as previously described [38]. The injection dose was 10.00 mg/kg, and the day before the operation, a Shikonin 1 ml syringe was used to administer 0.05 ml/10 g/mouse by tail vein injection.

### Laboratory animals

The animals used in the experiment were adult male wild-type (WT) C57 B6J mice (6–8 weeks of age; weight: 20–25 g) provided by the Experimental Animal Center of the Third Military Medical University (Chongqing, China). Adult male EGLN3^CKI/CKI, Cdh5−creERT2^ and PKM2^CKI/CKI, Aldh1| 1−creERT2^ mice (aged 6–8 weeks; weight: 20–25 g) were purchased from Cyagen Biotech Co., Ltd., USA. The method used for the generation of the transgenic mice is shown in Fig. 7A. The two kinds of mice were raised in the animal room of the Central Laboratory of Southwest Hospital, Third Military Medical University. The animals were caged and kept at a constant temperature, constant humidity, and a 12 h/12 h day/night cycle and were given sufficient water and food. The animals were randomly assigned to each experimental group according to the random number table method before intervention. All animal experimental protocols were approved by the Third Military Medical University Experimental Animal Ethics Committee (AMUWEC2020793), in line with the requirements of the ARRIVE 2.0 guidelines. The number of animals used for each experiment and the total number of animals used are detailed in the Supplementary Material.

### SAH model

To eliminate the effect of estrogen on the pathophysiology following subarachnoid hemorrhage, mice randomly assigned to the experimental group were deprived of food 12 h prior to the experiment. The SAH mouse model was established by the internal carotid artery suture method [39]. Mice were anesthetized using gas anesthesia (anesthetic gas was an air gas mixture containing 1.5% isoflurane). After the skin on the necks of the mice was shaved and disinfected, a median incision was made, followed by isolation of the external carotid artery. After making an incision approximately 2 mm above the bifurcation of the common carotid artery, 5–0 sharp monofilament nylon suture plugs were inserted into the external carotid artery and ligated distal to the incision. The thread bolt was reversed into the internal carotid artery through the common carotid artery bifurcation, and then the thread shirt was inserted upward through the internal carotid artery approximately 1 cm. The respiratory rate of the mice was observed until it changed, and then the thread was advanced 2 mm. Then, the thread bolt was removed, and the mice were ligated and sutured. A mouse subarachnoid hemorrhage model was established by this method. The sham operation group underwent the same procedure as the operation group except that the vessel was not perforated. To ensure the stability of the subarachnoid hemorrhage model, mice that died during or after surgery and those with a skull base hemorrhage score less than 8 were not used in this study.

### Plasmids

The three plasmids used in the in vitro experiments were all provided by Chongqing Jinmai Biotechnology Co., Ltd. (1) The pcDNA3.1 (+) plasmid vector was used for the overexpression of EGLN3 and PKM2. (2) The PSD 1211-U6-shRNA vector was used for PKM2 interference. In the in vitro experiments, 2 μl of Lipofectamine™ 2000 (11668500, Invitrogen) was mixed with 4 μg of DNA and allowed to return to room temperature for 20 min. After removing the culture medium, the cells were washed with culture medium once; then, 800 μl of culture medium and 200 μl of DNA transfection mixture were added to each well of the six-well plate, and the cells were transfected for 6 h. After the supernatant was removed, 2 ml of culture medium was added to each well, and the transfection was verified after 72 h.

### Neurological assessment

The modified Garcia score was measured on an 18-point scale, and the mice were evaluated for 6 indicators at 1 day, 3 days and 7 days after SAH, with a total score of 3 points for each indicator, for a total of 18 points. The 6 indicators that were evaluated were autonomous movement, posture symmetry, forelimb extension movement, grasping and cage climbing ability, bilateral body tactile reflex and bilateral beard tactile response. The balance beam test method refers to the methods used in a previous experiment [40].

### Open field experiment

The sham-operated group and the mice on days 1, 3 and 7 after SAH were placed into Behavior Atlas 3D behavioral equipment. The experimental device was a 100 × 100 cm square with sidewalls. The camera above the experimental site recorded the animals in real time throughout the entire experimental process. For each mouse, 5 min of video data were collected, and the trajectory and speed of each mouse were analyzed. The movement distance of each mouse within 5 min was determined for statistical analysis to evaluate motor ability.

### Brain water content experiment

The sham-operated group and the mice 1 day, 3 days and 7 days after surgery were anesthetized and decapitated to remove the brain tissue. The brain tissue was weighed on an electronic balance, and the weight was recorded. Then, the brain tissue was left in a 50 °C oven for 48 h. The brain was reweighed, and the brain water content was determined using the following equation: brain water content = 1 − (weight before drying-weight after drying) × 100%. The results were statistically analyzed.

### SAH bleeding score

According to a previous study [40, 41], the degree of hemorrhage in mice after SAH was assessed using an 18-point skull base hemorrhage scale. The lateral cranial base of the brain of mice after SAH was divided into 6 regions, and the degree of hemorrhage was scored from 0 to 3 according to the area of hemorrhage in each region. The scores of the 6 regions were added to generate the SAH hemorrhage score. Mice with a score of 7 or less were considered to have too low a degree of hemorrhage and were not used for further experiments.

### Transmission electron microscopy (TEM)

The mice were anesthetized and perfused with 0.9% normal saline through the left ventricle to the right atrium and then decapitated to remove the brain tissue. A small piece of tissue with a volume of 1 mm^3^ was placed in a 4% glutaraldehyde solution at 4 °C for 24 h. The brain tissue was then fixed in epoxy resin (Agar 100 resin, Agar Scientific). Observations were performed with a transmission electron microscope (JEM-1200 EX, JEOL) after staining with toluidine.

### Evans blue leakage

As our previous study [41], mice were anesthetized and injected with 2% Evans blue (DK 0051, LEAGENE) via the tail vein at a dose of 5 ml/kg, and blue coloration of the skin tissue was observed after 1 h of SPF culture. Mice were anesthetized and perfused with 0.9% normal saline through the left ventricle to the right atrium, followed by 4% paraformaldehyde. The brain tissue was removed, and after the same fixation method as was used for immunofluorescence frozen sections, coronal section (30 μm) were made and observed via confocal microscopy using the autofluorescence of Evans blue at an excitation wavelength of 555 (Olympus OX 51, Tokyo, Japan). For the absolute quantification of Evans blue, the brains of anesthetized mice were perfused with 0.9% normal saline through the left ventricle to the right atrium, and the brain tissues were removed by decapitation and homogenized in methylformamide (1 ml/100 mg brain tissue). After centrifugation at 200 g/min, the supernatant was removed, and the samples were left at 50 °C for 48 h; then, the OD value was determined by a microplate reader at 620 nm.

### Preparation of primary astrocytes from mouse brains

Within 1 day of birth, the suckling mice were artificially sacrificed, the brain tissue was removed after disinfection, the meninges were removed under an operating microscope, and the cerebral cortex tissue was isolated. The brain tissue was minced using a sterile instrument in 0.25% trypsin with EDTA (25200072, Gibco™) and left at 37 °C for 5 min. Then, an appropriate amount of 40 μg/ml DNase I (DN25, Sigma‒Aldrich) was added to digest the DNA. The resulting cell suspension was centrifuged at 400 × g for 5 min, and the supernatant was discarded. This procedure was repeated twice. The resulting cell pellet was resuspended in high-glucose DMEM (11965118, GibcoTM) supplemented with 10% fetal bovine serum and seeded in cell culture flasks. The cells were cultured in a 37 °C incubator containing 5% CO_2_ for 1 day, after which new media was added on day 5. After the cell coverage area reached more than 90%, the horizontal shaker was set to a speed of 200 rpm/min for 24 h, and the supernatant was removed. The obtained cells were used for subsequent cell experiments after the cell purity was determined to be > 99% by GFAP (NB 100-53809, NOVUS) immunofluorescence staining.

### Preparation of primary microvascular endothelial cells from mouse brain tissue

The brain tissue was cut and digested in HEPES (12,360,038, Gibco™) containing 30 U/ml papain (A501612, Sangon Biotech) and 40 μg/ml DNase I for 60 min as described above. At the end of digestion, 30% bovine serum albumin (A8010, Solarbio) was added to the PBS. The resulting cell suspension was centrifuged at 250 × g for 5 min, and the cells were resuspended in endothelial cell culture medium (CC-3202, Lonza) and plated in culture dishes precoated with 0.02% collagen type I (C8061, Solarbio). After 1 day of culture in a 37 °C cell incubator at 5% CO2, the medium was changed. The endothelial cells were passaged 3 times to purify the endothelial cells, and the cells were used for subsequent cell experiments after the purity of the cells was > 99%, as determined by immunofluorescence staining with CD 31 (1:100, NB100-2284, NOVUS).

### Transverse electrical resistance (TEER) measurement

Astrocytes were seeded in transwell chambers. When the cell density reached more than 90%, the TEER of the cells was measured using a Millicell ERS-2 resistograph (MERS 00002, Merck Millipore). The accuracy and stability of the measurements by the instrument were calibrated according to the instructions before measurement. The electrodes were inserted vertically into the transwell chamber during measurement. To ensure accuracy, the electrodes did not touch the sidewall or the bottom of the culture dish during measurement. The TEER value was calculated by subtracting the basal TEER value when there were no cells in the transwell chamber.

### Western blotting

Western blotting was performed according to previous studies [35]. Protein was extracted from the brain tissue on the hemorrhagic side using T-PER Tissue Protein Extraction Reagent (78510, Thermo Fisher Scientific). In the cellular experiments, the culture medium was removed, and the cell culture dish was washed with PBS. The lysate (P0013 K, Beyotime) was added to the cell culture dish (150–250 μl/10 cm^2^), and the cells were lysed on ice for 10 min and collected by a cell scraper. After centrifugation at 1000 × g for 10 min, the protein was collected. After the concentration was determined, 30 μg of sample was added to each well of an SDS‒PAGE gel, and the protein was transferred to a PVDF membrane after gel electrophoresis. The PVDF membrane was blocked with blocking solution for 2 h at room temperature, the blocking solution was washed with TBST, and the primary antibody dilution solution was added. After incubation at 4 °C for 24 h, the antibodies were washed with TBST. Horseradish peroxidase (HRP)-conjugated secondary antibody was selected according to the molecular weight of the target protein. The secondary antibodies were added to the PVDF membrane, and the membrane was incubated for at least 1.5 h at room temperature. A Fusion FX Edge Spectra multifunctional imaging system (Vilber Bio Imaging) was used for imaging and analysis. The antibodies and concentrations used in the experiments were HIF-1α (1:1000, NB100-105, NOVUS), EGLN3 (1:500, NB100-139, NOVUS), PKM2 (1:1000, NBP1-48308, NOVUS), PKC (1:1000, NB600-201, NOVUS), p-PKC (1:500, NBP3-21584, NOVUS), ERK (1:1000, AF1576, NOVUS), p-ERK (1:500, AF1018, NOVUS), and GAPDH (1:1000, NB300-221, NOVUS)**.**

### Coimmunoprecipitation assay

Protein was extracted from the cells by the method described in the previous section, and the protein concentration was determined. The primary antibody used for immunoprecipitation was added, and the sample was slowly shaken for 24 h in a 4 °C refrigerator. Then, 50 µl of fully resuspended protein A + G agarose was added, and the samples were slowly shaken for 2 h at 4 °C. Then, the sample was centrifuged at 1000 × g for 5 min at room temperature, and the supernatant was aspirated. After washing with PBS, the supernatant was removed, 30 µl of 1X SDS‒PAGE loading buffer was added, and electrophoresis was performed after incubating for 5 min in a 100 °C water bath. The proteins were then transferred to a PVDF membrane. The subsequent antibody incubation and development procedures were the same as those for Western blotting. The antibodies used in this study were EGLN3 (1:1000, NB100-139, NOVUS), PKM2 (1:1000, NBP1-48308, NOVUS), and IgG (2 μg, A7016, Beyotime) antibodies.

### Protein docking experiment

The 3D structures of EGLN3 and PKM2 were obtained from the AlphaFold and PDB public databases, and the protein information was analyzed by GRAMM (http://www.example.com). The docking results were analyzed by PyMOL software, and the protein docking structure map, specific docking site structure and molecular information were obtained (see supplementary dataset).

### Fluorescence immunohistochemistry and immunocytochemistry

The mice were anesthetized and perfused with 0.9% normal saline through the left ventricle to the right atrium; then, 4% paraformaldehyde was used for further perfusion. The brain tissue was removed and fixed in 4% paraformaldehyde for 24 h. The tissues were dehydrated in 30% sucrose PBS for 24 h, followed by dehydration in 40% sucrose/PBS solution for 2 days. The brain tissues were fixed with OCT and sectioned coronally at a thickness of 30 μm in a freezing microtome (CM3050S-3-1-1, Leica). The cells were incubated in PBS containing 0.5% Triton-100 for 10 min, washed with PBS, and then incubated in goat serum for 2 h. At 4 °C, an antibody diluent was added, and the samples were incubated for 24 h. The antibody diluent was washed off with PBS, and the diluent containing the corresponding secondary antibodies was added (the secondary antibodies were prepared in the dark). The samples were incubated at room temperature for 2 h. The slides were finally mounted in an anti-quenching mounting medium containing DAPI stain and visualized using a confocal microscope (LSM 880, Zeiss). For immunofluorescence staining of cells, the cells were passaged in confocal dishes, the medium was removed, the cells were washed with PBS after the corresponding treatments, and the cells were fixed with 4% paraformaldehyde for 30 min. The subsequent test method was the same as that for the immunofluorescence staining of frozen sections of brain tissue. Positive cells were counted, and the fluorescence intensity of the immunofluorescence staining results was measured using ImageJ V1.53 (National Institutes of Health). The antibodies and concentrations used in this experiment were as follows: claudin 1 (1:500, 37-4900, Invitrogen), connexin 43 (1:500, C6219-100, Sigma‒Aldrich), GFAP (1:800, NB100-53809, Novus), CD 31 (1:100, NB100-2284, NOVUS), ZO 1 (1:100, NBP 1-85046, NOVUS), CD45 (1:500, 70257S, Cell Signaling Technology), EGLN3 (1:200, NB100-139, NOVUS), PKM2 (1:200, NBP 1-48308, NOVUS) and vWF (1:200, NB600-586, NOVUS).

### Quantitative polymerase chain reaction (PCR)

Cells from conditional knock-in mice were added to 100 μl digestion solution and digested for 15 min in a metal bath at 55 °C, followed by a 5 min incubation in a metal bath at 95 °C and centrifugation; the supernatant was used for PCR. The specific method was performed as described previously [42]. The primer sequences are shown in Supplementary Table 1.

### Bioinformatics analysis

Bioinformatics data were collected from the GEO database, human brain microvascular endothelial cells were obtained 24 h after hypoxia treatment (GSE 163827), and human astrocytes after hypoxia treatment for 24 h (GSE 145935) were analyzed [43]. Difference analysis (heatmaps and volcano plots) and enrichment analysis (KEGG analysis) were carried out by R language and the corresponding program package. The requirements for a significant difference were logFc > 1 and P < 0.05.

### Flow cytometry

After anesthesia, the mice were perfused with PBS through the left ventricle to the right atrium and then decapitated to remove brain tissue. RPMI 1640 medium (11875093 GibcoTM) was added to Dounce, and the brain tissue was ground into a single-cell suspension and filtered through a 100 μm cell sieve. The NK cells and neutrophils were extracted using a flow cytometry kit (P2430, Solarbio) according to the manufacturer’s instructions. Briefly, the single-cell suspension was added to the prepared gradient centrifugation liquid, and the sample was centrifuged at 1000 × g for 30 min. The target cells were removed and resuspended in PBS containing 10% FBS, centrifuged at 250 × g for 10 min to wash the cells three times, resuspended in 100 μl of PBS containing 10% FBS and subjected to flow cytometry antibody staining. The specific antibodies used were FITC-conjugated CD45 (553079, BD), PE-conjugated NK1.1 (553165, BD), PerCP-CyTM5.5-conjugated CD3e (551163, BD), PE-CY 7-conjugated CD11b (552850, BD), and APC-conjugated LY-6G (560599). Flow cytometry was performed using an ACEA flow cytometer (Novcell), and the results were analyzed using Flow Jo v7.6 software (Informer Technologies).

### Statistical analysis

The data were analyzed and plotted with GraphPad Prism 7 and are presented as the means and SEMs. The normality of the data was tested using the Shapiro‒Wilk test, and homogeneity of variance was tested using the F test. Nonnormally distributed data were analyzed by nonparametric tests and the Kruskal‒Wallis test. The sample size of each group was greater than or equal to 3. Data from the immunofluorescence staining of wild-type mice and a series of experiments, including behavior assays, wild-type primary cell immunofluorescence staining, and Western blotting, were analyzed by one-way ANOVA. Data from the immunofluorescence staining of transgenic mice and wild-type mice after SAH and other experiments, including the immunofluorescence staining of transfected plasmid cells and wild-type primary cells, were analyzed by two-way ANOVA. We used post hoc Bonferroni-Dunn correction to compare the data between groups. A P value less than 0.05 was considered statistically significant.

## Results

### Significant time lag between endothelial barrier disruption and peak brain edema

**Fig. 2:**
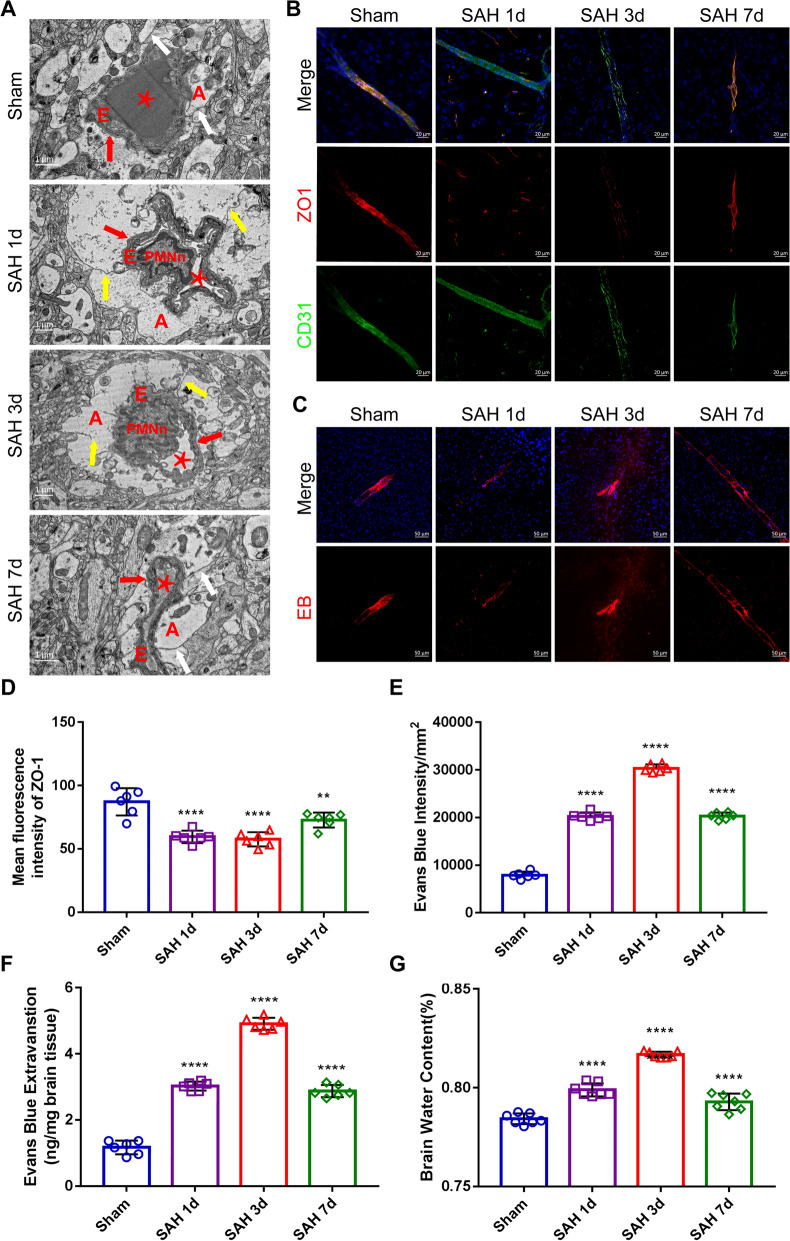
BBB disruption and cerebral edema after SAH. **A** TEM of cerebral microvasculature in the cortical region of C57BL/6 mice on days 1, 3 and 7 in the sham and SAH groups (scale bar, 1 μm). (A-astrocytes end-foot; E-endothelial cells; white arrows-gap junctions; yellow arrows-tight junctions; red arrows -vascular basement membrane; *-blood vessels; PMNn-polymorphonuclear neutrophils). **B** and** D** Immunofluorescence labeling of ZO1 (Zonula occludens protein 1, red), CD31 (platelet endothelial cell adhesion molecule-1, green), and DAPI (4ʹ,6-diamidino-2-phenylindole, blue) on days 1, 3 and 7 in the sham and SAH groups of C57BL/6 mice (scale bar, 50 μm). n = 6 per group; one-way ANOVA, **P < 0.01 vs. the sham group; ****P < 0.0001 vs. the sham group. **C, E **and** F** Representative images of Evans blue (red) autofluorescence and Evans blue extravasation on days 1, 3 and 7 in the sham and SAH groups of C57BL/6 mice (scale bar, 50 μm). n = 6 per group; one-way ANOVA; ****P < 0.0001 vs. the sham group. **G** Brain water content was measured on days 1, 3 and 7 in the sham and SAH groups of C57BL/6 mice. n = 8/7, one-way ANOVA, **P < 0.01 vs. the sham group; ****P < 0.0001 vs. the sham group

First, we observed by transmission electron microscopy that endothelial cells were structurally disorganized and that tight junctions were disrupted 1 day after SAH (Fig. 2A); subsequently, we examined the expression of ZO-1 at BBB tight junctions over time after SAH. Immunofluorescence staining revealed that disruption of tight junctions occurred on day 1, and this relatively severe degree of damage persisted until day 3, with recovery occurring only after day 7 (Fig. 2B and D). To further validate the disruption of blood‒brain barrier function, we used semiquantitative and quantitative methods of EB autofluorescence and found that although the BBB was destroyed on the first day after SAH and was comparable to the typical BBB on the third day, EB exudation was most severe on the third day (Fig. 2C, E and F). This phenomenon was also confirmed in the brain water content test (Fig. 2G). The above experimental results corroborate the clinical paradox that exists; it was stated above that there is a significant time lag between the disruption of the tight junctions between the endothelium and the peak of brain edema.

### BBB disruption after SAH is accompanied by the formation of GLs around blood vessels

**Fig. 3:**
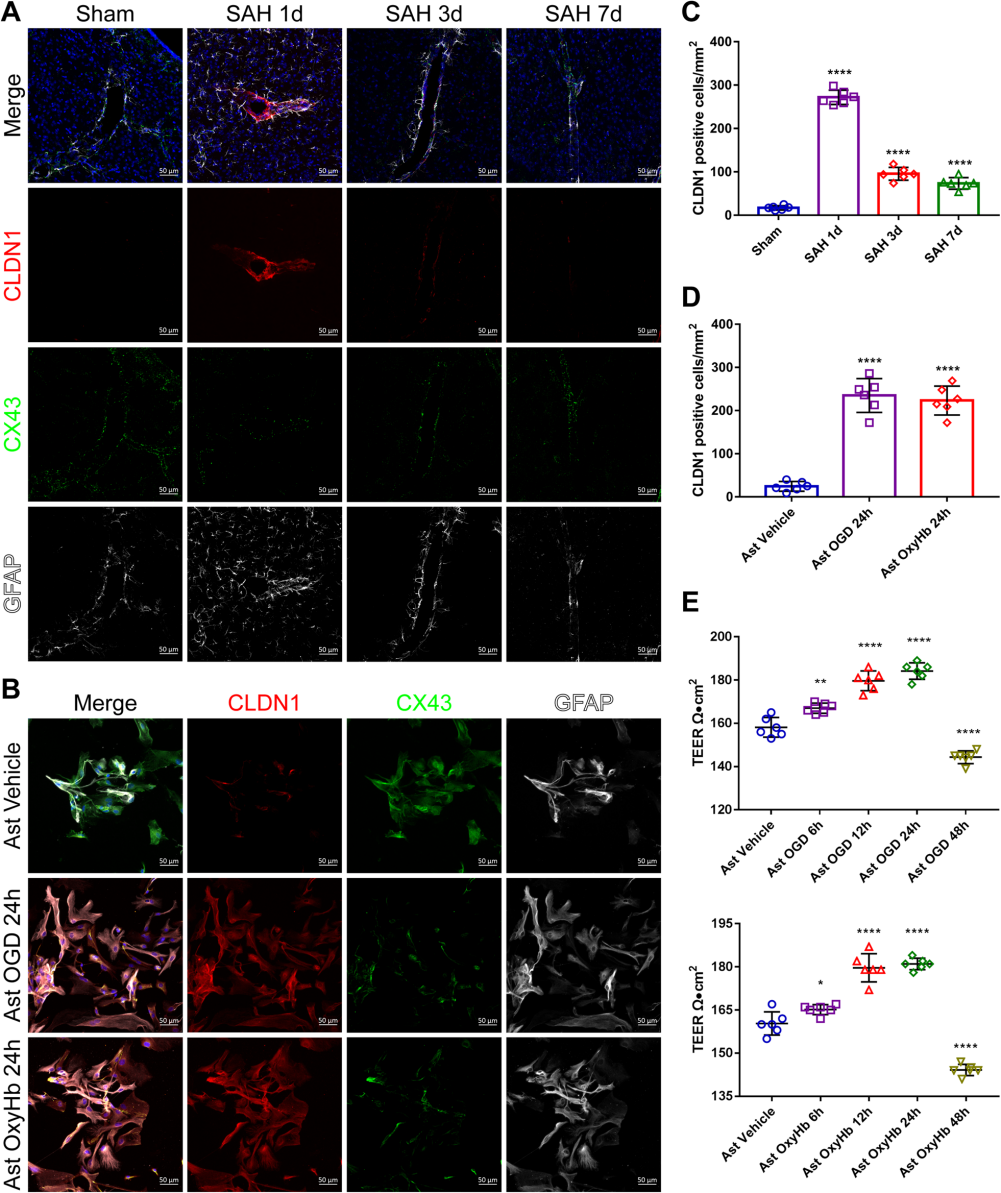
Spatial changes and the localization of GL structures after SAH. **A **and** C** Immunofluorescence labeling of CLDN1 (claudin-1, red), CX43 (connexin 43, green), GFAP (glial fibrillary acidic protein, white), and DAPI (blue) on days 1, 3 and 7 in the sham and SAH groups of C57BL/6 mice (scale bar, 50 μm). CLDN1 colocalized with GFAP. n = 6 per group; one-way ANOVA; ****P < 0.0001 vs. the sham group. **B** and** D** Immunofluorescence labeling of CLDN1 (red), CX43 (green), GFAP (white) and DAPI (blue) after the treatment of primary astrocytes with OGD and OxyHb (scale bar, 50 μm). n = 6 per group; one-way ANOVA; ****P < 0.0001 vs. the astrocyte vehicle group. **E** TEER values of the astrocyte Vehicle group, the OGD-treated group and the OxyHb intervention group after 6 h, 12 h, 24 h and 48 h. n = 6 per group, 1-way ANOVA, **P < 0.01 vs. the ast vehicle group; ***P < 0.001 vs. the ast vehicle group; ****P < 0.0001 vs. the ast vehicle group

Previous studies and our experiments (Supplemental Fig. S1) demonstrated that astrocyte end feet wrap around blood vessels and may form GLs after SAH to act as a barrier. Ultrastructural observation of the BBB by transmission electron microscopy revealed that the astrocyte end feet were significantly swollen, the number of tight junctions was increased 1 day after SAH, and the end feet cell membranes were damaged and ruptured with the weakening of tight junctions 3 days later (Fig. 2A). By immunofluorescence staining, we found that under physiological conditions, the expression level of CLDN1 was very low, and there was no obvious aggregation. CX43 was densely expressed in astrocyte end feet. The expression of CLDN1 significantly increased around the BBB 1 day after SAH and gradually decreased after 3 and 7 days. At the same time, the change in the expression of CX43 was opposite to that of CLDN1, and CX43 expression obviously decreased 1 day after SAH and gradually recovered after 3 days and 7 days (Fig. 3A and C). This finding suggested that astrocytes form a protective barrier after SAH, especially on the first day, but that barrier function weakens with increasing time. In addition, CLDN1 expression was significantly upregulated in cultured primary astrocytes after glucose deprivation (OGD) and oxygen and hemoglobin (OxyHb) treatment for 24 h (Fig. 3B and D). In addition, the TEER of cultured primary astrocytes began to increase 6 h after OGD and OxyHb treatment, peaked at 24 h, and decreased significantly at 48 h (Fig. 3E), which also confirmed the results of the in vivo experiments. Through these experiments, we confirmed that astrocytes can form temporary tight junction structures at the same time as endothelial cells are destroyed after SAH, which may have a protective effect.

### GLs help maintain BBB function during the acute phase after SAH

**Fig. 4:**
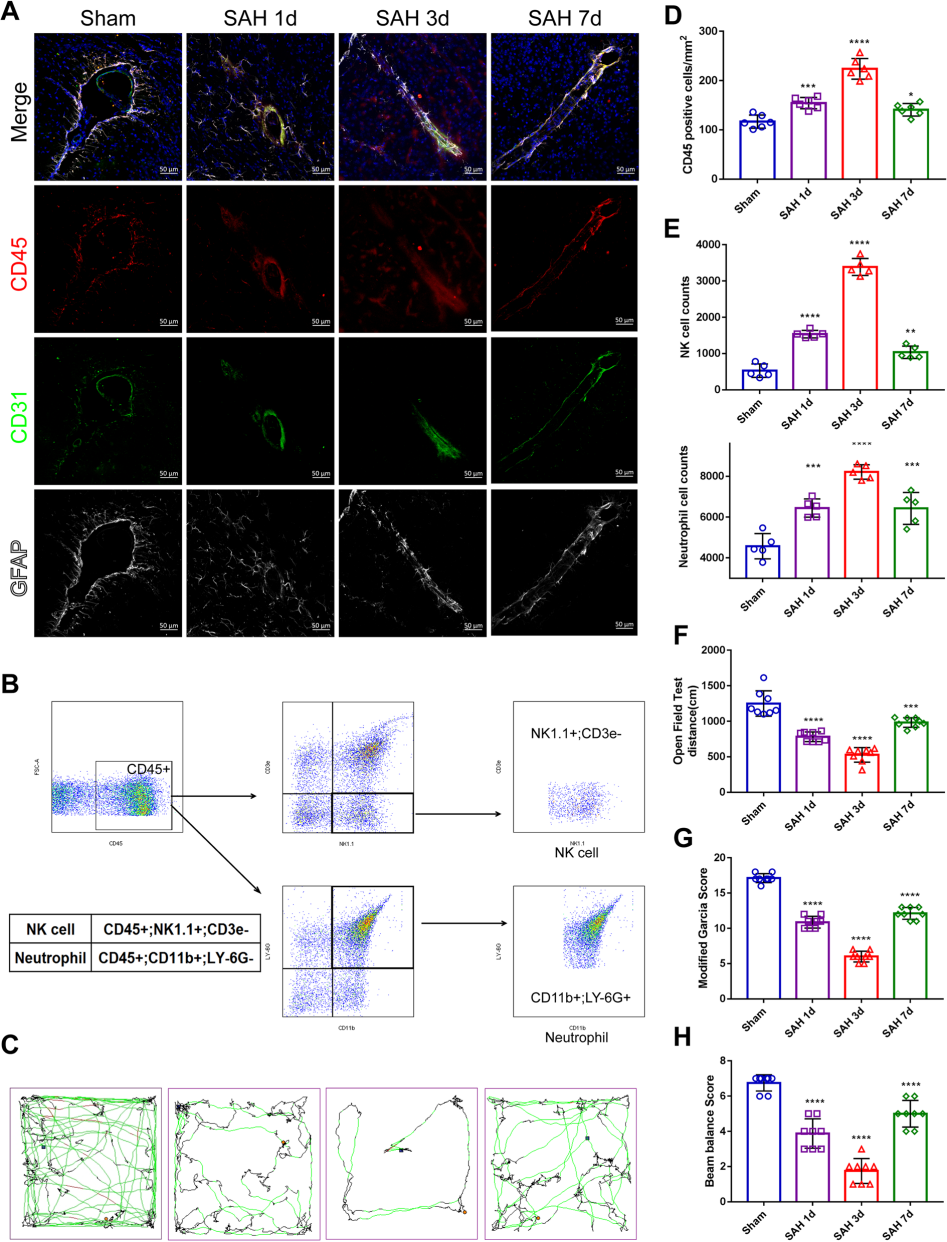
GL barrier function and the effect on neurological outcomes after SAH. **A **and** D** Immunofluorescence labeling of CD45 (leukocyte common antigen, red), CD31 (green), GFAP (white) and DAPI (blue) on days 1, 3 and 7 in the sham and SAH groups of C57BL/6 mice (scale bar, 50 μm). n = 6 per group; one-way ANOVA, *P < 0.05 vs. the sham group; ***P < 0.001 vs. the sham group; ****P < 0.0001 vs. the sham group. **B** and** E** Flow cytometry plots and quantification showing the expression of infiltrating inflammatory cells, including NK cells and neutrophils, obtained from brain tissue on days 1, 3 and 7 in the sham and SAH groups of C57BL/6 mice (scale bar, 50 μm). n = 6 per group; one-way ANOVA, **P < 0.01 vs. the sham group; ***P < 0.001 vs. the sham group; ****P < 0.0001 vs. the sham group. **C **and** F** Open field test. The movement trajectories and the quantitative statistics of distances on days 1, 3 and 7 in the sham and SAH groups of C57BL/6 mice. The mice underwent the training test on the open field 24 h before the test. n = 8; one-way ANOVA, ***P < 0.001 vs. the sham group; ****P < 0.0001 vs. the sham group. **G** and** H** Modified Garcia scores (**G**) and beam balance scores (**H**) were measured on days 1, 3 and 7 in the sham and SAH groups of C57BL/6 mice. n = 8/7, one-way ANOVA, **P < 0.01 vs. the sham group; ****P < 0.0001 vs. the sham group

To investigate whether GLs act as a barrier to protect the brain from inflammatory infiltration and other injuries, immunofluorescence staining and flow cytometry analysis of CD45-positive cells were performed, which revealed that CD45-positive cells (Fig. 4A and D), neutrophils (CD45 + ; CD11b + ; Ly-6 G +) and NK cells (CD45 + ; NK 1. 1 + ; CD 3-) (Fig. 4B and E) appeared 1 day after SAH, and their levels increased significantly at 3 days, suggesting that GLs in the acute phase after SAH do have a barrier function to some extent to reduce vascular permeability and protect the central nervous system from injury.

Next, we confirmed the role of GLs in protecting against neurological damage after SAH. In the open field test, the movement distance of mice after SAH was significantly reduced, and the impairment of motor ability appeared on the first day, was most severe on the third day, and recovered on the seventh day (Fig. 4C and F). The same results were obtained for the modified Garcia score and balance beam test (Fig. 4G and H). These results correspond to the changes in GLs over time, further indicating that GLs can protect neurological function, reduce brain edema, and improve prognosis in mice after SAH; these effects are limited to the acute stage and decrease over time.

### Bioinformatics analysis revealed that EGLN3 in endometrial cells is a potential switch that regulates the astrocyte response to injury

**Fig. 5:**
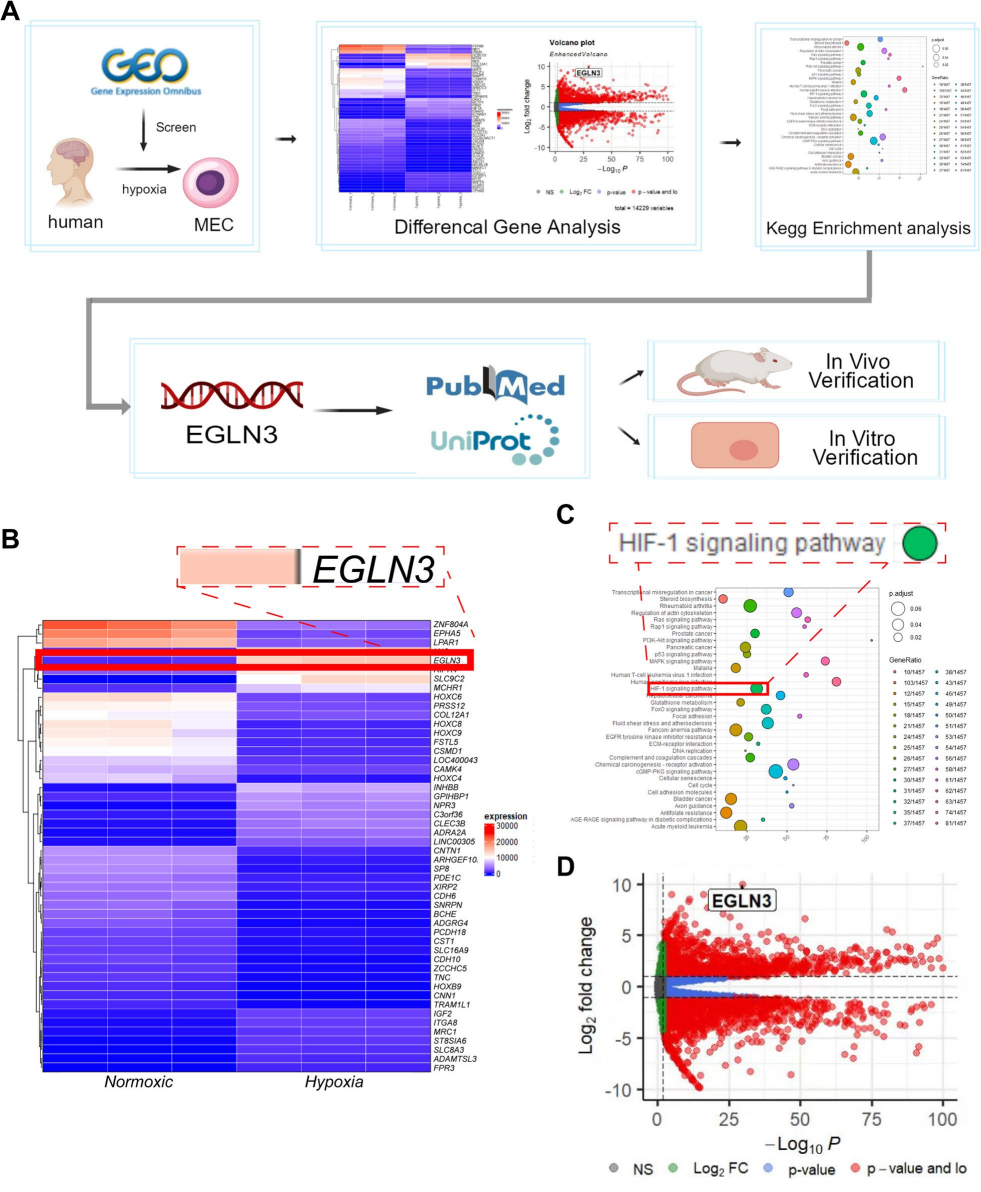
Bioinformatic analysis of microvascular endothelial cell RNA-seq transcriptomes. **A** The bioinformatics analysis of microvascular endothelial cells included data selection, data analysis, gene screening and experimental verification. **B** Heatmap showing the top 50 proteins related to hypoxia in microvascular endothelial cells and the location of the EGLN3 gene. **C** KEGG pathway enrichment analysis showed that the Hif1 alpha signaling pathway and the MAPK signaling pathway were closely related to hypoxia. **D** Volcano plot showing the significantly differentially expressed genes identified between microvascular endothelial cells in the normoxia and hypoxia groups and the location of the EGLN3 gene

Next, we explored the factors that influence the formation of GLs after SAH. Previous immunofluorescence staining and electron microscopy results (Fig. 2A) revealed that the astrocyte endfoot tightly wraps vascular endothelial cells, and many previous studies have shown that endothelial cells have an important impact on astrocytes after SAH. Therefore, we collected RNA-seq transcriptome data from human brain microvascular endothelial cells under hypoxic conditions from the GEO database for bioinformatics analysis (Fig. 5A). We first screened for differentially expressed genes in endothelial cells after hypoxia using heatmaps (Fig. 5B), and then we performed KEGG analysis of differentially expressed genes (Fig. 5C). The HIF-1α signaling pathway was most strongly correlated with hypoxia and had the most gene enrichment. Among these genes, the gene with the most upregulated expression was EGLN3, and previous studies have also shown that EGLN3 is strongly correlated with hypoxia (insert literature). We further demonstrated the association of EGLN3 with hypoxia using volcano plots (Fig. 5D).

### Endothelial cells perceive hypoxic injury stimuli and upregulate EGLN3

**Fig. 6:**
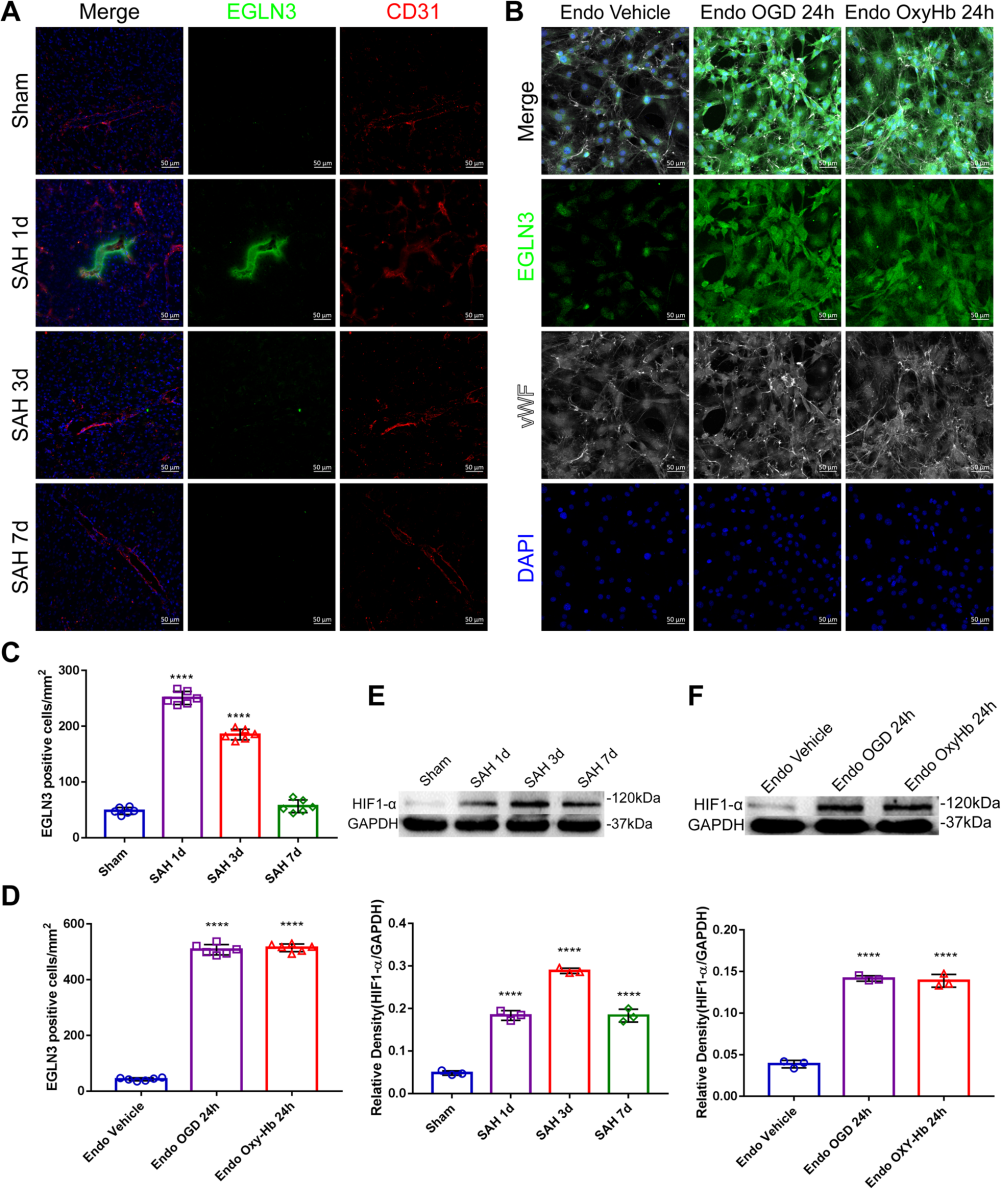
Changes in EGLN3 expression in endothelial cells after SAH. **A **and **C** Immunofluorescence labeling of EGLN3 (prolyl hydroxylase EGLN3, green), CD31 (red), and DAPI (blue) on days 1, 3 and 7 in the sham and SAH groups of C57BL/6 mice (scale bar, 50 μm). n = 6 per group; one-way ANOVA; ****P < 0.0001 vs. the sham group. **B **and** D** Immunofluorescence labeling of EGLN3 (green), vWF (von Willebrand factor, white), and DAPI (blue) in the endothelial cell vehicle group, the OxyHb intervention group and the OGD-treated group after 24 h. n = 6 per group, 1-way ANOVA, ****P < 0.0001 vs. the Endo vehicle group. **E** and **F** Western blotting and quantitative analysis of Hif1-alpha on days 1, 3 and 7 in the sham and SAH groups of C57BL/6 mice and the endothelial cell vehicle group, the OxyHb intervention group and the OGD-treated group after 24 h. n = 3 per group, 1-way ANOVA, ****P < 0.0001 vs. the sham group

We verified the results of the bioinformatics analysis and found that EGLN3 expression was significantly upregulated in endothelial cells 1 day after SAH and gradually decreased at 3 and 7 days with the development of hemorrhage (Fig. 6A and C); this change was similar to that in GLs, which was most obvious on the first day and gradually decreased thereafter. Moreover, in vitro experiments revealed that the expression of EGLN3 was also significantly increased in cultured primary endothelial cells 24 h after OxyHb treatment and OGD treatment (Fig. 6B and D). Western blotting analysis of the HIF-1α signaling pathway revealed that HIF-1α expression was significantly increased 1 day after SAH but gradually decreased 3 days and 7 days after SAH (Fig. 6E). In vitro, the expression of HIF-1α was also significantly increased in primary endothelial cells 24 h after OGD treatment and OxyHb treatment (Fig. 6F). These results confirmed the results of the bioinformatics analysis of endothelial cells, and the expression of EGLN3 in endothelial cells was closely related to SAH.

### PKM2 is regulated in astrocytes after SAH and is influenced by endothelial cells

**Fig. 7:**
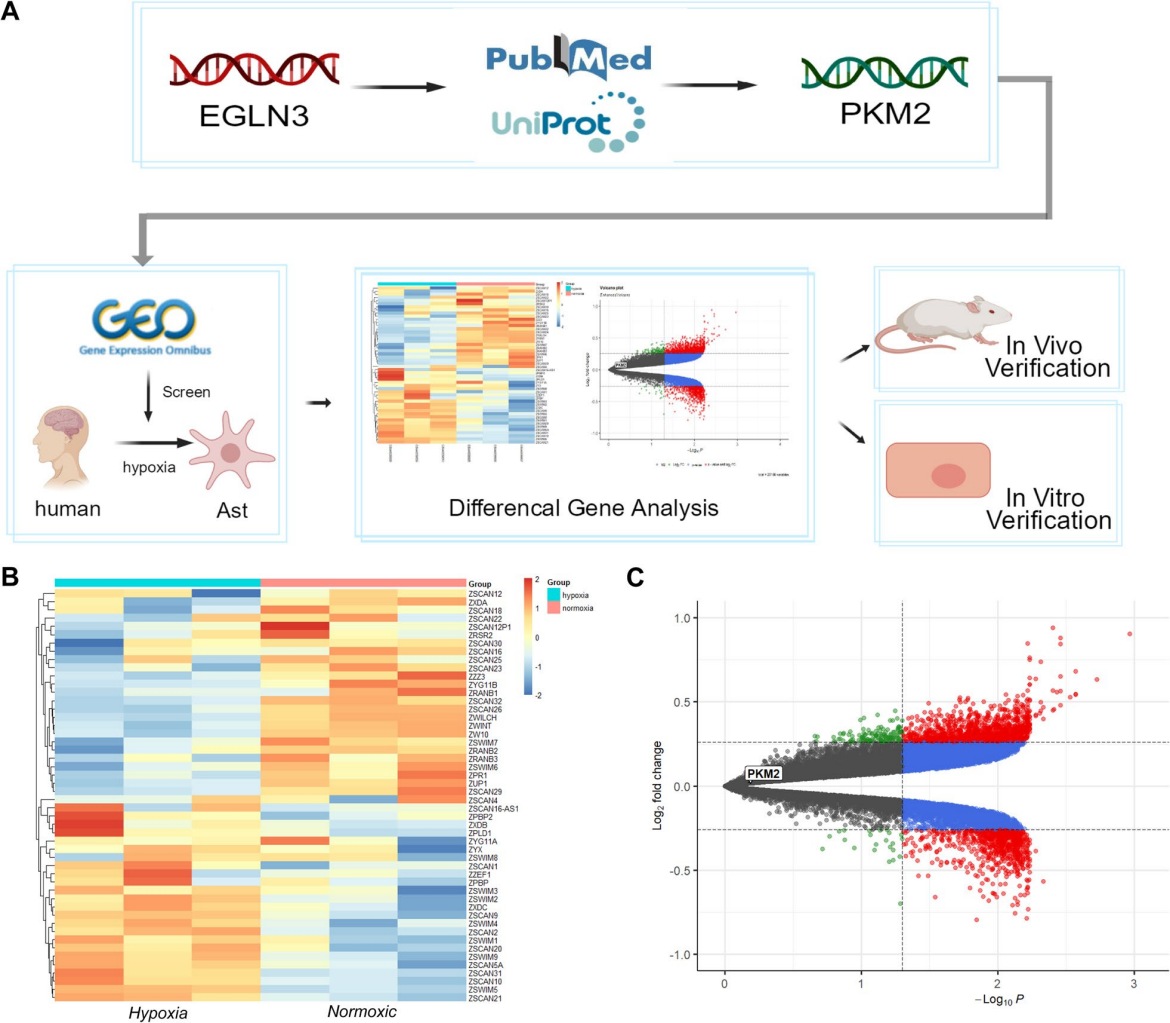
Bioinformatics analysis of the transcriptomes of human astrocytes. **A** The bioinformatics analysis of astrocytes included data selection, data analysis, gene screening and experimental verification. **B** Heatmap showing the top 50 proteins related to hypoxia in astrocytes. **C** Volcano plot showing the significantly differentially expressed genes identified when comparing the normoxia and hypoxia groups of astrocytes and the location of the PKM2 gene

UniProt and previous studies have shown that EGLN3 is closely related to PKM2 [24]. EGLN3-mediated hydroxylation of PKM2 is a sensor of cell stress caused by hypoxia and injury. Therefore, we next utilized transcriptomic data from human astrocytes under hypoxia from the GEO database for bioinformatics analysis (Fig. 7A). Interestingly, the heatmap and volcano plot of differentially expressed genes showed that PKM2 expression was not upregulated in astrocytes after hypoxia treatment (Fig. 7B and C). This result was different from that of previous studies [24], but data from GEO database experiments involving in vitro culture and the treatment of a single type of cell did not include interactions between cells; thus, we hypothesized that after SAH in astrocytes, PKM2 may be activated by endothelial cell EGLN3 signaling and promote the formation of GLs.

**Fig. 8:**
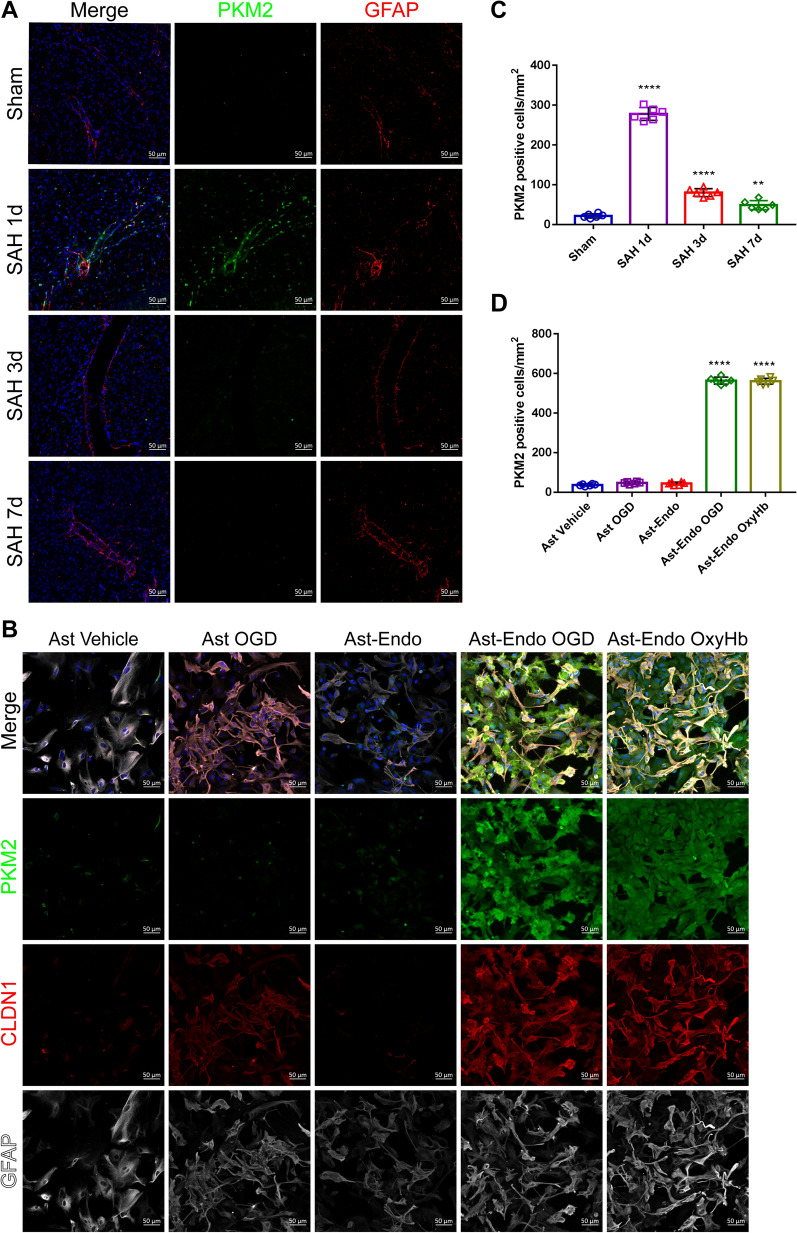
Changes in PKM2 expression in astrocytes after SAH. **A **and** C** Immunofluorescence labeling of PKM2 (pyruvate kinase M2, green), GFAP (red), and DAPI (blue) on days 1, 3 and 7 in the sham and SAH groups of C57BL/6 mice (scale bar, 50 μm). n = 6 per group; one-way ANOVA, **P < 0.01 vs. the sham group; ****P < 0.0001 vs. the sham group. **B** and** D** Immunofluorescence labeling of PKM2 (green), CLDN1 (red), GFAP (white), and DAPI (blue) in the astrocyte vehicle group, the astrocyte OGD group, the ast-endo medium group, the ast-endo OGD medium group and the ast-endo OxyHb medium group after 24 h. n = 6 per group, 1-way ANOVA, ****P < 0.0001 vs. the ast vehicle group

To verify the changes in PKM2 expression in astrocytes and to determine whether these changes are related to EGLN3, we performed immunofluorescence staining and found that PKM2 expression in astrocytes was significantly upregulated on the first day after SAH and then gradually decreased (Fig. 8A and C). To verify whether PKM2 upregulation is associated with endothelial cells, we designed five sets of in vitro cell experiments (Supplemental Fig. S2). Interestingly, PKM2 expression was not upregulated in astrocytes treated with OGD alone, which was consistent with the bioinformatics analysis results. However, PKM2 expression was significantly upregulated in glial cells treated with endothelial medium. More importantly, CLDN1 expression was significantly greater in astrocytes in the ast-endo OGD medium group and the ast-endo OxyHb medium group than in astrocytes in the vehicle group and the ast-endo medium group (Fig. 8B and D), indicating that PKM2 activation is closely related to the formation of CLDN1. This finding explains why the upregulation of PKM2 expression in astrocytes after SAH involves endothelial cells and is likely mediated by EGLN3.

### Endothelial cell-derived EGLN3 activates PKM2 in astrocytes after SAH

**Fig. 9:**
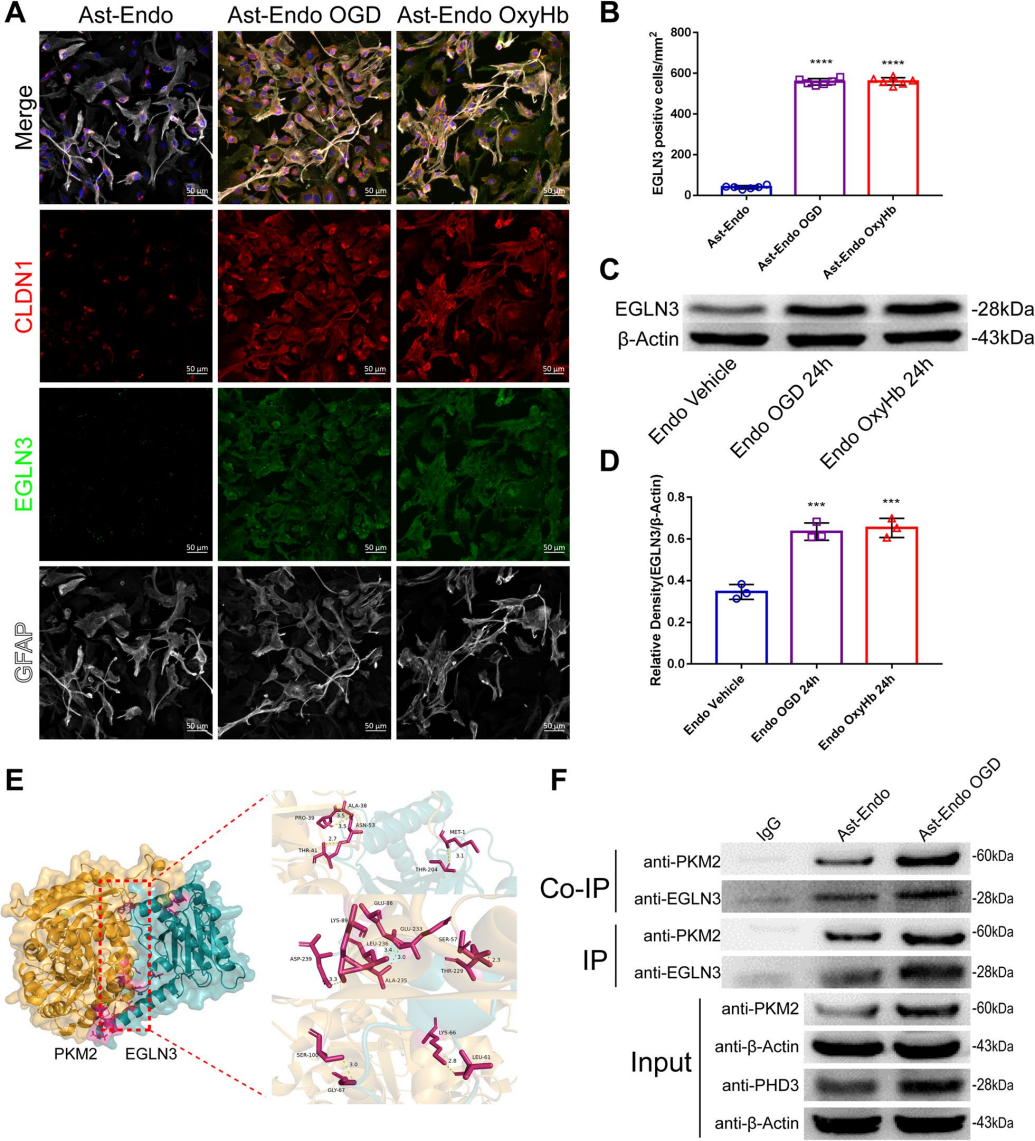
Changes in EGLN3 in astrocytes after SAH and the correlation between EGLN3 and PKM2. **A** and** B** Immunofluorescence labeling of CLDN1 (red), EGLN3 (green), GFAP (white), and DAPI (blue) in the ast-endo medium group, the ast-endo OGD medium group and the ast-endo OxyHb medium group after 24 h. n = 6 per group, 1-way ANOVA, ****P < 0.0001 vs. Ast Vehicle group. **C** and** D** Western blotting and quantitative analysis of EGLN3 in the cell supernatants of the vehicle group, the OGD-treated group and the OxyHb intervention group after 24 h. n = 3 per group, 1-way ANOVA, ****P < 0.0001 vs. the sham group. **E** The results of process docking, the three-dimensional structure of PKM2 (yellow) and EGLN3 (blue) process docking and docking sites. **F** The protein interactions were analyzed by coimmunoprophylaxis. The interaction between EGLN3 and PKM2 was verified by forward and reverse experiments

We further verified that PKM2 in astrocytes is affected by EGLN3 derived from endothelial cells by immunofluorescence, which showed that EGLN3 was significantly increased in astrocytes treated with OGD and OxyHb-treated endothelial cell culture medium (Fig. 9A and B). In addition, Western blot analysis of endothelial cell culture medium revealed that the EGLN3 level in the culture medium of OGD- and OxyHb-treated endothelial cells was significantly greater than that in the culture medium of vehicle-treated endothelial cells (Fig. 9C and D). This finding suggested that EGLN3 in astrocytes is derived from endothelial cells. To determine whether there was an interaction between proteins, we first used protein docking (Fig. 9E), and the analysis results showed that a very stable docking structure can be formed between EGLN3 and PKM2 (see Supplemental Dataset for specific docking sites). Furthermore, to determine whether EGLN3 coprecipitated with the PKM2 antibody or the EGLN3 antibody, a coimmunoprecipitation experiment was performed, and the results showed that there was a strong interaction between the two proteins (Fig. 9F). In conclusion, EGLN-3 signaling in endothelial cells affects PKM-2 expression in astrocytes after SAH and thus may affect CLDN1 expression and promote GL formation.

### EGLN3 regulates CLDN1 expression through PKM2 after SAH

**Fig. 10:**
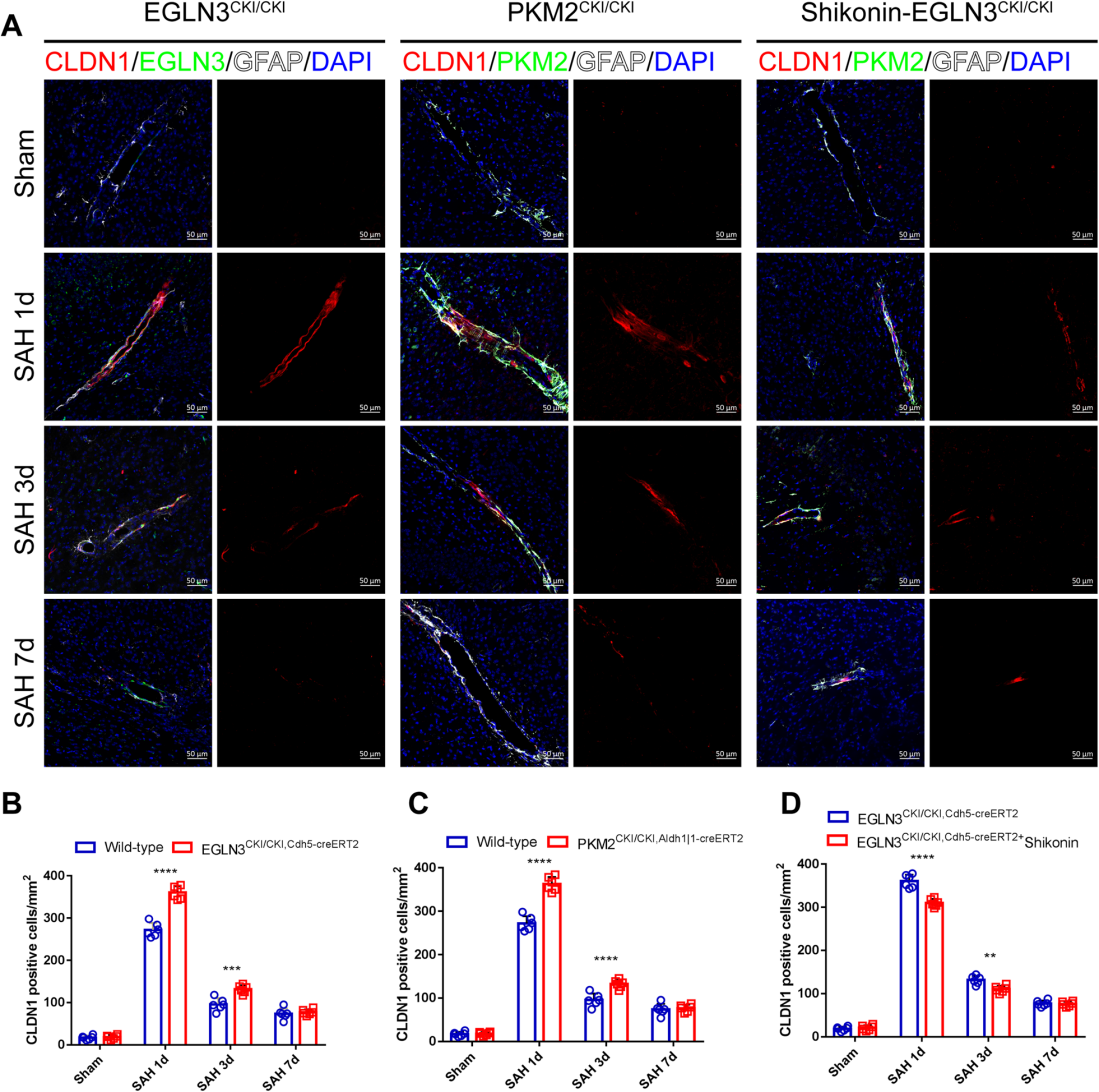
EGLN3 or PKM2 could enhance the tight junction expression of GL in vivo. **A–D** Immunofluorescence labeling of CLDN1 (red), EGLN3 (green), GFAP (white) and DAPI (blue) on days 1, 3 and 7 in the sham and SAH groups of WT mice and EGLN3^CKI/CKI, Cdh5−creERT2^ mice; Immunofluorescence labeling of CLDN1 (red), PKM2 (green), GFAP (white) and DAPI (blue) on days 1, 3 and 7 in the sham and SAH groups of WT mice, PKM2^CKI/CKI, Aldh1| 1−creERT2^ mice and Shikonin-treated EGLN3^CKI/CKI, Cdh5−creERT2^ mice (scale bar, 50 μm). n = 6 per group; two-way ANOVA, ***P < 0.001; ****P < 0.0001

Next, we examined whether the upregulation of CLDN1 expression after SAH was associated with the upregulation of EGLN3 expression in endothelial cells and PKM2 expression in astrocytes, and most importantly, we examined whether the effect of EGLN3 on CLDN1 involved PKM2. To test our hypothesis, we generated transgenic mice with conditional knock-in of EGLN3 and PKM2 (Supplemental Fig. S3) and verified successful knock-in of the target genes by PCR (Supplemental Fig. S4). Immunofluorescence staining revealed that the expression of CLDN1 in EGLN3^CKI/CKI, Cdh5−creERT2^ mice and PKM2^CKI/CKI, Aldh1|1−creERT2^ mice (Fig. 10A–C) after SAH was still strongest on the first day after SAH and then gradually decreased. Interestingly, compared with that in wild-type mice, the expression of CLDN1 was significantly increased on the first and third days after SAH, and there was no significant difference on the seventh day. Next, we used Shikonin (a known inhibitor of PKM2) to inhibit PKM2 expression in EGLN3^CKI/CKI and Cdh5−creERT2^ mice and verified its inhibition by Western blotting (Supplementary Fig. S5). The results showed that CLDN1 expression significantly decreased on days 1 and 3 after SAH compared with that in EGLN3^CKI/CKI and Cdh5−creERT2^ mice without the inhibitor. There was no significant difference on the seventh day (Fig. 10A and D). These results suggest that PKM2 plays an important role in the EGLN3-mediated upregulation of CLDN1 expression.

**Fig. 11:**
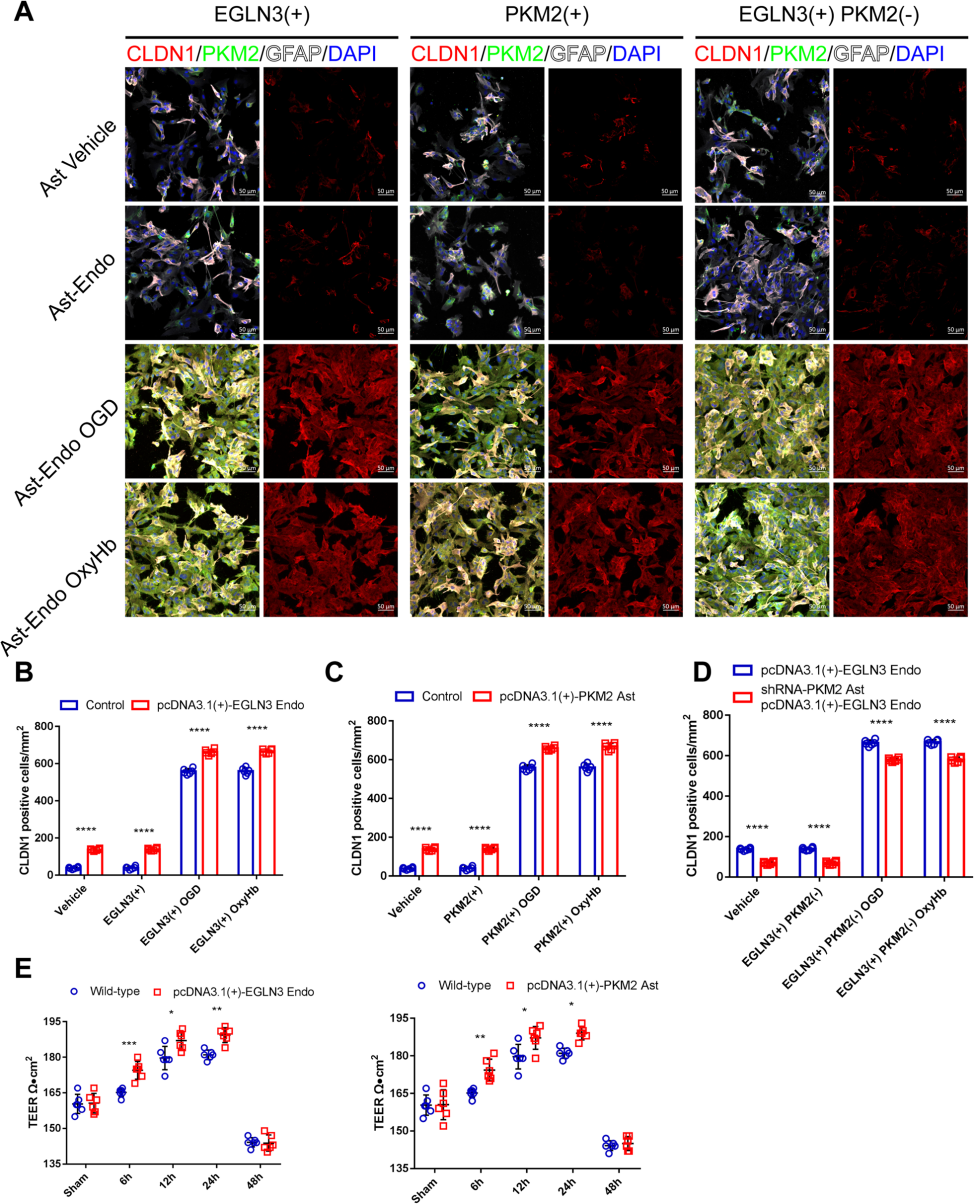
EGLN3 or PKM2 could enhance the tight junction expression of GL in vitro. **A–D** Immunofluorescence labeling of PKM2 (green), CLDN1 (red), and GFAP (white) and DAPI (blue). The following four types of cells were used: the vehicle group (astrocytes were not treated); the EGLN3(+) group (endothelial cells were transfected with a pcDNA3.1(+)-EGLN3 plasmid); the PKM2(+) group (astrocytes were transfected with a pcDNA3.1(+)-PKM2 plasmid); and the EGLN3(+) PKM2(−) group (astrocytes were transfected with a Psd1211-U6-shRNA-PKM2 plasmid, and endothelial cells were transfected with a pcDNA3.1(+)-EGLN3 plasmid). The cells were treated in the following four different ways: astrocytes were not treated, astrocytes were cultured with endothelial cell culture medium, astrocytes were cultured with OGD-treated endothelial cell culture medium, and astrocytes were cultured with OxyHb-treated endothelial cell culture medium. Immunofluorescence staining was performed 24 h later. n = 6 per group; two-way ANOVA; ****P < 0.0001. **E** TEER values were measured. The following three types of cells were used: the WT group (astrocytes were not treated), the pcDNA3.1(+)-EGLN3 endothelial group (endothelial cells were transfected with a pcDNA3.1(+)-EGLN3 plasmid) and the pcDNA3.1(+)-PKM2 astrocyte group (astrocytes were transfected with a pcDNA3.1(+)-PKM2 plasmid). The cells were treated in the following two different ways: astrocytes were cultured with OGD-treated endothelial cell culture medium, and astrocytes were cultured with OxyHb-treated endothelial cell culture medium for 0 h, 6 h, 12 h, 24 h or 48 h. n = 6 per group, 2-way ANOVA, *P < 0.05; **P < 0.01; ***P < 0.001

In in vivo experiments, changes in protein expression in cells were achieved by plasmid transfection and verified by Western blotting (Supplementary Fig. S6). Immunofluorescence staining revealed that the pcDNA3.1 (+)-EGLN3 plasmid caused endothelial cells to overexpress EGLN3. After OxyHb and OGD intervention (the culture method is shown in Supplemental Fig. S2) in the same astrocyte cell type, the endothelial cell culture medium after simulated SAH treatment upregulated the expression of CLDN1 in astrocytes. More importantly, in an experiment in which EGLN3 was overexpressed in endothelial cells using plasmids, the expression of CLDN1 in astrocytes was significantly upregulated compared with that in the wild-type endothelial cell medium group (Fig. 11A and B). The same result was obtained in the experiment using the pcDNA3.1 (+)-PKM2 plasmid, which caused astrocytes to overexpress PKM2 (Fig. 11A and C). Furthermore, similar to the in vivo results, after replacing wild-type astrocytes cultured with pcDNA3.1 (+)-EGLN3 endothelial cell medium with medium from astrocytes transfected with the Psd1211-U6-shRNA-PKM2 plasmid, CLDN1 expression was still upregulated after OxyHb and OGD treatment, but CLDN1 expression was significantly downregulated in Psd1211-U6-shRNA-PKM2 astrocytes compared with that in wild-type astrocytes (Fig. 11A and D). In the TEER experiments, the upregulation of EGLN3 expression in endothelial cells and PKM2 expression in astrocytes resulted in a significant increase in the TEER of astrocytes starting 6 h after the indicated treatments and lasting for at least 24 h (Fig. 11E). According to these results, after SAH, endothelial cells activate EGLN-3 signaling through PKM-2 in astrocytes and then promote the expression of CLDN1 to promote the formation of GLs.

### EGLN3 and PKM2 upregulate the expression of CLDN1 to improve the barrier function of GLs

**Fig. 12:**
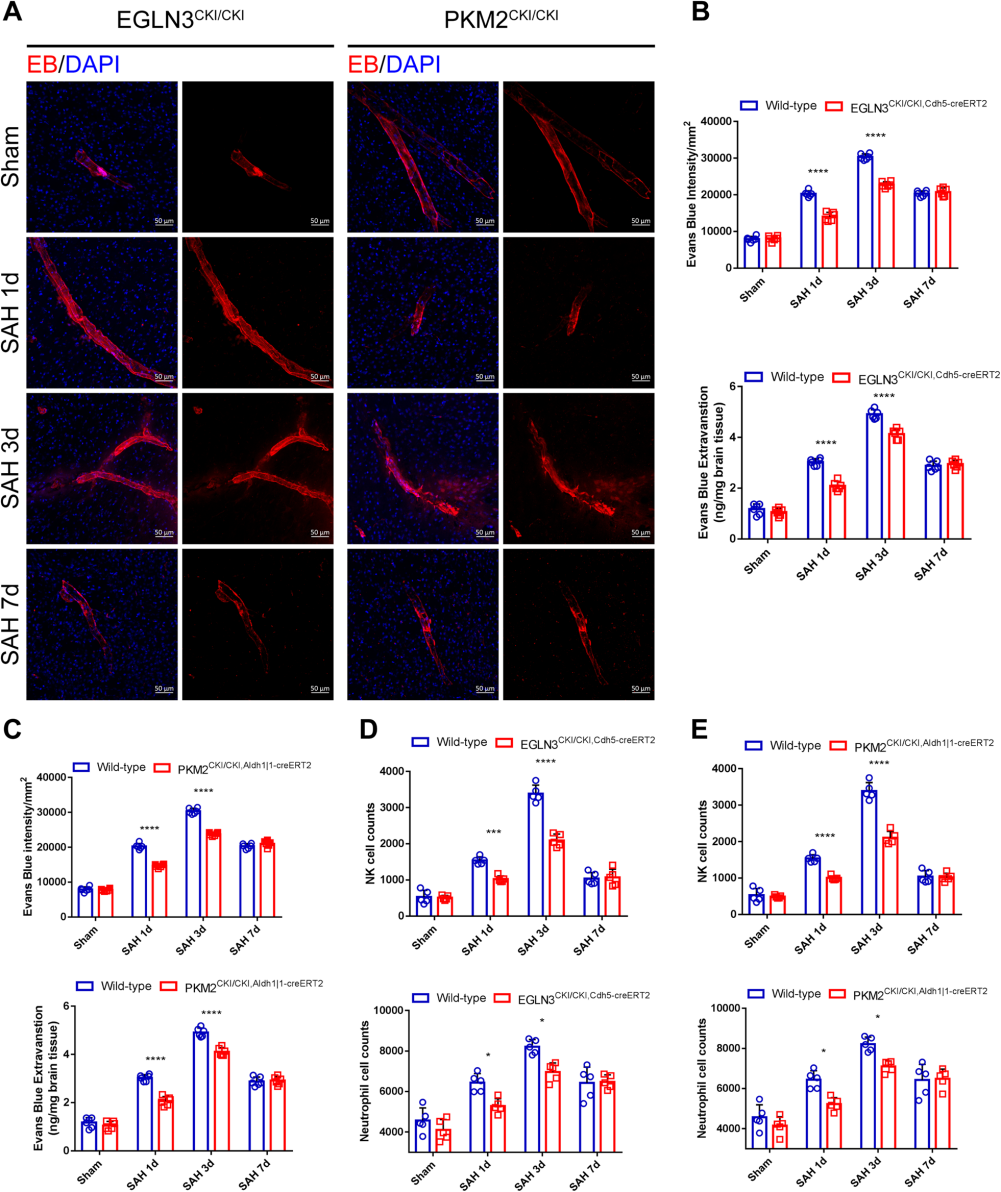
EGLN3 and PKM2 maintain the barrier function of the BBB. **A–C** Representative images of Evans blue (red) autofluorescence and Evans blue extravasation on days 1, 3 and 7 in the sham and SAH groups of WT mice, EGLN3CKI/CKI, Cdh5-creERT2 mice and PKM2CKI/CKI, Aldh1| 1-creERT2 mice (scale bar, 50 μm). n = 6 per group; two-way ANOVA; ****P < 0.0001. **D** and** E** Flow cytometry quantification showing the expression of infiltrating inflammatory cells, including neutrophils and NK cells, obtained from brain tissue on days 1, 3 and 7 in the sham and SAH groups of WT mice, EGLN3CKI/CKI, Cdh5-creERT2 mice, PKM2CKI/CKI mice, and Aldh1| 1-creERT2 mice (scale bar, 50 μm). n = 6 per group, 2-way ANOVA, *P < 0.05 vs. sham group; ***P < 0.001 vs. sham group; ****P < 0.0001

We aimed to confirm whether the improvements in GL function mediated by EGLN3 and PKM2 can protect the central nervous system from damage. Through Evans blue autofluorescence and fluorescence semiquantitative analysis of EGLN3^CKI/CKI, Cdh5−creERT2^ and PKM2^CKI/CKI, Aldh1|1−creERT2^ mouse brain tissues after SAH, we found that the upregulation of EGLN3 and PKM2 expression enhanced the barrier function of GLs and reduced Evans blue extravasation in the brain parenchyma on days 1 and 3 after SAH, but there was no significant effect on day 7 (Fig. 12A and B). The same results were obtained in the Evans blue absolute quantification assay (Fig. 12C). We also found that the upregulated expression of EGLN3 and PKM2 reduced the infiltration of NK cells and neutrophils in the brain parenchyma 1 and 3 days after SAH, but there was no significant difference at 7 days compared with that in the wild-type group (Fig. 12D and E). Therefore, we confirmed that the upregulation of CLDN1 expression mediated by EGLN3 and PKM2 could enhance the barrier function of GLs, thus enhancing CL-mediated protection of the central nervous system after SAH.

### Upregulation of EGLN3 and PKM2 improves neurological prognosis and reduces cerebral edema after SAH

**Fig. 13:**
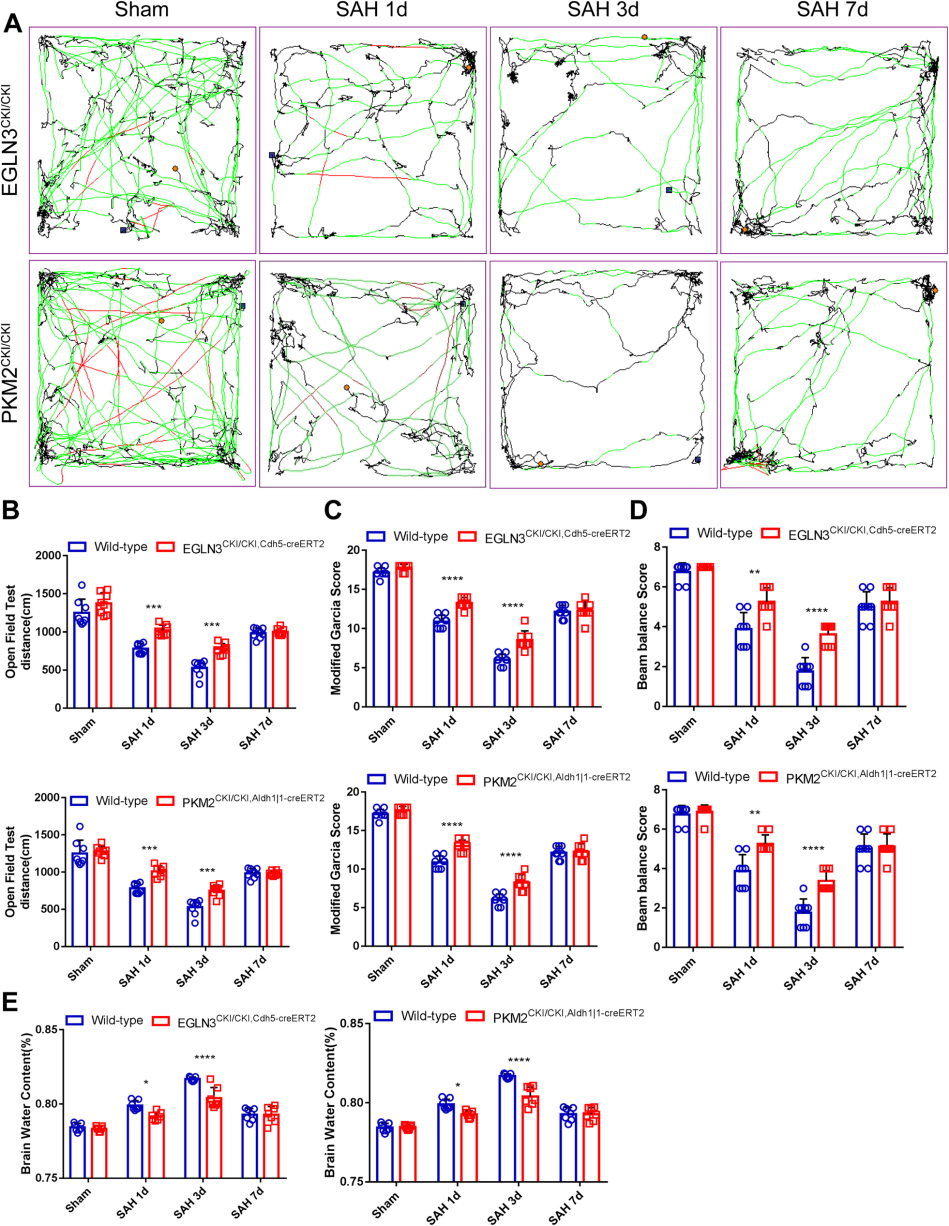
Protective effects of EGLN3 and PKM2 on the brain parenchyma and neural function. **A** and** B** Open field test. The movement trajectories and the quantitative statistics of distances on days 1, 3 and 7 in the sham and SAH groups of WT, EGLN3CKI/CKI, Cdh5-creERT2, PKM2CKI/CKI, and Aldh1| 1-creERT2 mice. The mice underwent the training test in the open field 24 h before the test; n = 8 per group; two-way ANOVA, ***P < 0.001. **C–E** The modified Garcia score (**C**), beam balance score (**D**) and brain water content (**D**) were measured on days 1, 3 and 7 in the sham and SAH groups of WT, EGLN3CKI/CKI, Cdh5-creERT2, PKM2CKI/CKI, and Aldh1| 1-creERT2 mice. n = 8 per group, 2-way ANOVA, *P < 0.05, **P < 0.01; ****P < 0.0001

Next, we investigated whether the upregulation of EGLN3 and PKM2 expression could improve the prognosis of mice after SAH. Through the open field test, we found that EGLN3^CKI/CKI, Cdh5−creERT2^ and PKM2^CKI/CKI, Aldh1|1−creERT2^ (Fig. 13A and B) mice had significantly increased movement distances on day 1 and day 3 after SAH, but there was no significant difference between Egln3, PKm2, and WT mice on day 7, indicating that the motor function of the mice was improved. Similarly, the modified Garcia scores of the EGLN3^CKI/CKI, Cdh5−creERT2^ and PKM2^CKI/CKI, Aldh1|1−creERT2^ mice (Fig. 13C) and their scores from the balance beam experiment (Fig. 13D) were significantly greater than those of the WT mice at 1 and 3 days after SAH, and there was no significant difference at 7 days. Moreover, the brain water content of the EGLN3^CKI/CKI, Cdh5−creERT2^ and PKM2^CKI/CKI, Aldh1|1−creERT2^ mice was significantly lower than that of the WT mice 1 and 3 days after SAH (Fig. 13E). According to these experimental results, the upregulation of EGLN3 and PKM2 improved the prognosis of mice after SAH by protecting against brain parenchymal infiltration and injury.

### PKM2 may affect the expression of CLDN1 via phonological PKC and activation of the MAPK signaling pathway

**Fig. 14:**
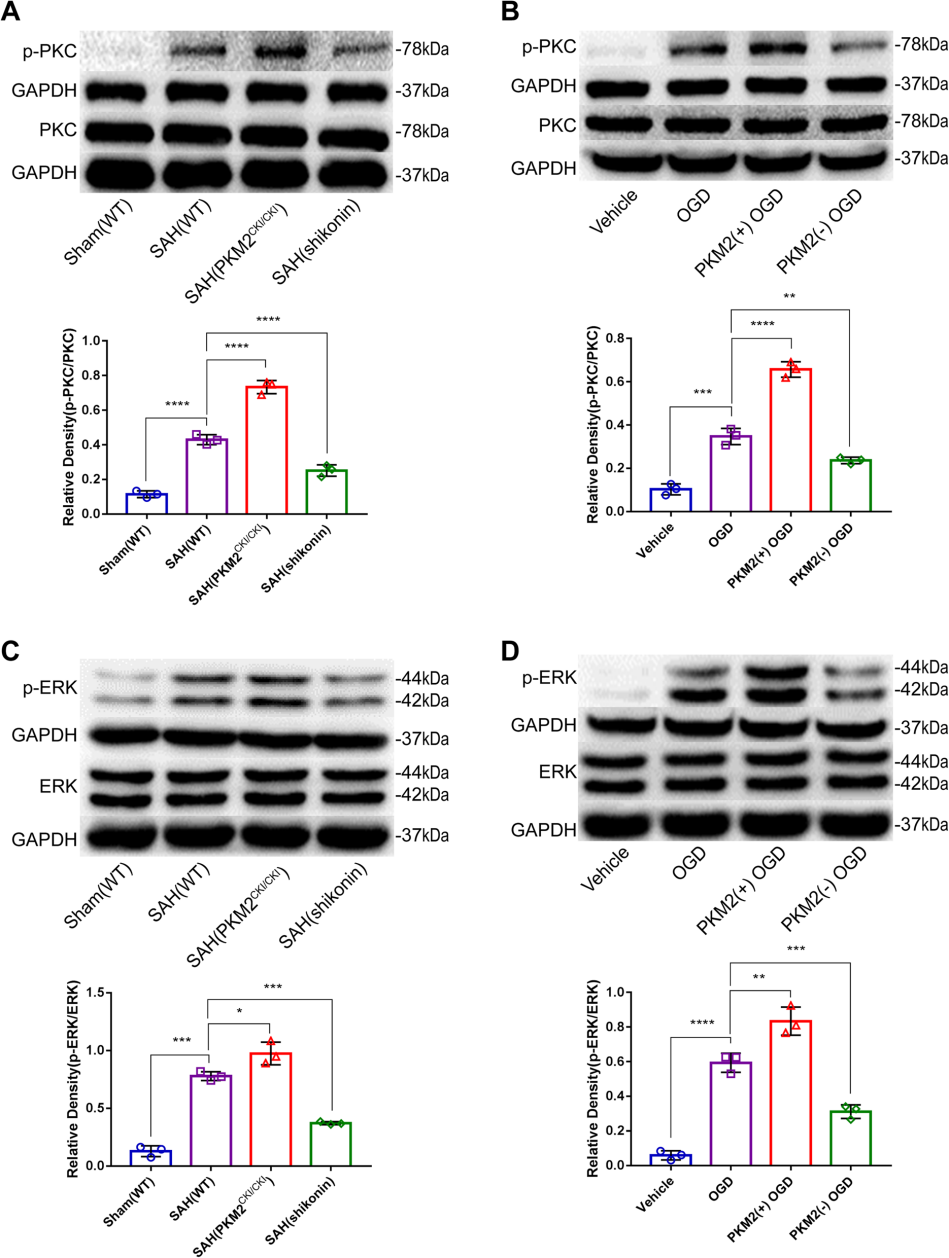
Expression of proteins downstream of PKM2 after SAH. **A–D** Western blotting and quantitative analysis of p-PKC, PKC, p-ERK and ERK. The following four kinds of mice were used for the in vivo study: WT sham mice and WT mice 24 h after SAH; PKM2CKI/CKI and Aldh1| 1-creERT2 mice 24 h after SAH; and WT mice 24 h after SAH. The cell grouping method was the same as that described in Sect. 3.6 (n = 3 per group); one-way ANOVA, *P < 0.05; **P < 0.01; ***P < 0.001; ****P < 0.0001

Finally, we investigated the possible signaling pathways by which PKM2 affects CLDN1 expression. Previous studies have shown that PKM2 may upregulate CLDN1 expression by phosphorylating PKC and subsequently activating the ERK/MAPK signaling pathway [36]. Moreover, we showed that the ERK/MAPK signaling pathway was enriched after hypoxia (Fig. 5C). Therefore, we investigated the changes in the protein expression of PKC, p-PKC, ERK and p-ERK on the first day after SAH by Western blotting. We found that PKC (Fig. 14A) expression did not change significantly in WT mice, PKM2^CKI/CKI, Aldh1|1−creERT2^ mice and Shikonin-injected WT mice after SAH, but p-PKC expression was significantly upregulated in WT mice, PKM2^CKI/CKI, Aldh1|1−creERT2^ mice and Shikonin-injected WT mice after SAH compared to healthy WT mice. Notably, the expression of p-PKC increased in PKM2^CKI/CKI, Aldh1|1−creERT2^ mice but decreased in Shikonin-treated WT mice compared to that in WT mice after SAH.

Furthermore, in the in vivo experiment, the supernatant of endothelial cells treated with OGD for 24 h was removed, and WT astrocytes, cDNA3.1(+)-PKM2 astrocytes and Psd1211-U6-shRNA-PKM2 astrocytes were added for another 24 h. The Western blotting results showed that there was no significant change in the expression of PKC (Fig. 14B), but the expression of p-PKC was significantly increased in the other three groups of cells compared with that in astrocytes without endothelial medium. Moreover, compared with that in WT astrocytes, the expression of p-PKC in pcDNA3.1(+)-PKM2-expressing astrocytes was increased, while that in Psd1211-U6-shRNA-PKM2-expressing astrocytes was decreased. Similar results were obtained for the changes in ERK and p-ERK expression in brain tissues and astrocytes in vitro (Fig. 14C and D). In conclusion, PKM2 may upregulate the expression of CLDN by phosphorylating PKC and subsequently activating the ERK/MAPK pathway after SAH.

## Discussion

**Fig. 15:**
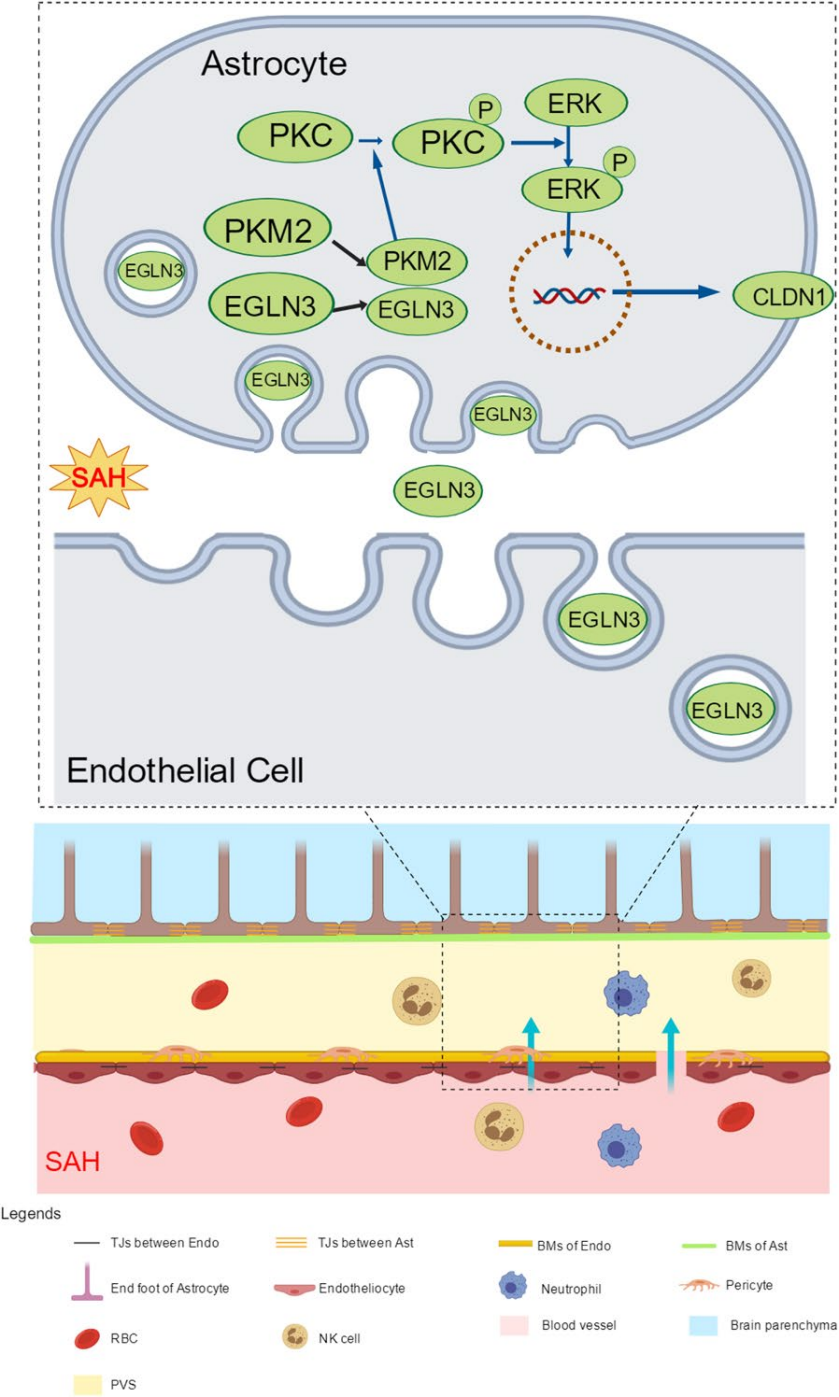
Schematic illustration of the present study. After SAH, tight junctions between endothelial cells are disrupted, but endothelial EGLN3-astrocyte PKM2 signaling promotes the formation of barrier-GLs between astrocytes, which reduces vascular leakage into the brain parenchyma, improves neurological prognosis, and attenuates the development of brain edema

In this study, we demonstrated (Fig. 15) that although the disruption of tight junctions between endothelial cells leads to BBB damage after SAH, astrocytes can form secondary barriers, called GLs, in an emergency manner early after SAH and prevent the infiltration of inflammatory cells, including neutrophils and NK cells, into the brain parenchyma, thereby reducing brain edema and protecting against neurological impairment. Furthermore, endothelial cells can activate PKM2 in astrocytes by increasing EGLN3 secretion after injury and then upregulate CLDN1 expression through activation of the PKC-ERK-MAPK pathway to enhance GL-mediated protection of the central nervous system. This may be the reason for the time difference between brain edema after SAH and BBB endothelial cell damage. In addition, the upregulated expression of EGLN3, a hypoxia sensor, can also activate the HIF-1α pathway, which generates a cellular stress response to hypoxia and thus prevents hypoxic injury.

The barrier function of astrocytes is a more general stress protection mechanism in the central nervous system [44]. An increasing number of studies have shown that in a variety of central nervous system injuries, including experimental autoimmune encephalomyelitis, repetitive traumatic brain injury and optic nerve injury [45, 46], GLs formed by astrocytes generally occur at the injury site [47] and are closely related to brain parenchymal inflammatory injury and neuronal migration errors [48, 49]. These results suggest that GLs may play an important role in central nervous system injury after SAH. This study and previous studies have further demonstrated that astrocyte endfeet are wrapped outside endothelial cells in a composite BBB structure [50]. Immunofluorescence staining and transmission electron microscopy showed that gap junctions are dominant between astrocyte endfeet in healthy states. On the first day after SAH, although the tight junctions between endothelial cells were damaged, the number of tight junctions between astrocyte endfeet (CLDN1) was increased, resulting in the formation of GLs similar to those of endothelial cells, which gradually disappeared on the 3rd and 7th days with the progression of injury and the rupture of the astrocyte cell membrane. Further studies revealed that GLs can prevent inflammatory cells from infiltrating the brain parenchyma after SAH and reduce brain edema and neurological dysfunction after SAH. This conclusion has also been confirmed in previous studies [51].

In previous studies, several important proteins and pathways, including the G-protein-coupled receptor (GPCR) Gpr 124, were found to activate the WNT pathway to repair the BBB following damage [52]. The concept of the neurovascular unit was proposed, and further research has shown that the real BBB includes a complex system that involves endothelial cells, astrocytes, pericytes and neurons [53], which together regulate the integrity of the BBB and protect the central nervous system [23]. More importantly, this protective effect is achieved through close interactions between endothelial cells and other cells in the neurovascular unit. For example, signaling communication between endothelial cells and pericytes helps maintain the integrity of the BBB [54, 55]. Similarly, there is evidence of an interaction between astrocytes and endothelial cells, which is key to protecting the central nervous system [23]. Alvarez, J. I. reported that astrocytes maintain the homeostasis of the BBB by secreting Sonic hedgehog and binding to the hedgehog receptor expressed on the endothelial cells of the BBB in healthy and diseased states [56]. Therefore, to investigate the effect of endothelial cells on GLs after SAH, we collected RNA-seq transcriptomic data of human brain microvascular endothelial cells after hypoxic treatment from the GEO database for bioinformatics analysis. Through differential gene expression and enrichment analyses, we found that the HIF-1α signaling pathway was enriched after hypoxia, and the upregulation of EGLN3 gene expression in this signaling pathway was likely related to the stress response of endothelial cells after SAH. EGLN3 is a hypoxic sensor and plays an important role in cancer and diabetes [57, 58]. PKM2 is a substrate of the proline hydroxylase EGLN3 [24]; therefore, we searched the GEO database for bioinformatics data on human astrocytes subjected to hypoxia but found that PKM2 expression was not upregulated in astrocytes subjected to hypoxia. However, as stated in the results section, this is inconsistent with previous studies [24]. The experimental data from the GEO database are related to in vitro culture and treatment of a single type of cell, not to cell‒cell interactions. In our study, we first verified the upregulation of EGLN3 expression in endothelial cells, PKM2 expression in astrocytes, and CLDN1 expression after SAH in vivo and in vitro. We then demonstrated that the upregulation of PKM2 and CLDN1 expression in astrocytes was affected by the high expression of EGLN3 in endothelial cells by Western blotting and coimmunoprecipitation of an endothelial cell-astrocyte culture system.

EGLN3/PKM2 signaling has been shown to help cells respond to hypoxic stress. Researchers discovered that EGLN3 can induce PKM2 prolyl hydroxylation, which amplifies the HIF1-alpha signal [34]. Further studies have shown that EGLN3 can form a complex with PKM2 and bind to heterogeneous nuclear ribonucleoprotein F (hnRNPF) to enter the nucleus and upregulate the transcription of HIF-1α [59]. EGLN3^CKI/CKI, Cdh5−creERT2^ and PKM2^CKI/CKI, Aldh1|1−creERT2^ mice were used as SAH models, and compared with WT mice, both EGLN3 and PKM2 upregulated CLDN1 expression, but the use of Shikonin to inhibit PKM2 downregulated the expression of CLDN1. Thus, EGLN-3 in endothelial cells affects PKM-2 and CLDN1 expression in astrocytes. To verify whether EGLN-3/PKM-2 signaling enhances the barrier effect on GLs and reduces brain edema and neurological dysfunction after SAH, we used flow cytometry, the Evans blue test, and behavioral and neurological scoring methods and obtained positive results. Moreover, inhibition of protein kinase C in colon cancer tumor cells (PKC) can cause a PKM2-mediated decrease in claudin-1 expression [36], and numerous studies have shown that PKC can upregulate CLDN1 expression [60, 61]. PKC/MAPK has been shown to be an important signaling pathway in multiple organs [62–64], and more importantly, consistent with the upregulation of CLDN1 expression in intestinal epithelial cells by PKC/MAPK signaling [37], the MAPK signaling pathway was significantly enriched in our bioinformatics analysis. Finally, we demonstrated by Western blotting that PKM2 upregulated CLDN1 expression through the PKC/ERK/MAPK signaling pathway.

We found that after SAH, astrocytes formed GLs to protect against BBB injury. In this process, EGLN3/PKM2 signaling between endothelial cells and astrocytes plays an important role, and increased PKM2 promotes the formation of GLs through the PKC/ERK/MAPK signaling pathway. However, the current study has several limitations. First, the time span of this study included only the acute phase one week after SAH, and the results of this study prove that the effect of GLs is limited to 1 day after SAH. Future studies should focus on determining how to prolong the presence of GLs to improve acute and even long-term prognoses after SAH. In addition, determining the specific molecular mechanism by which EGLN-3 acts on PKM-2 to promote CLDN1 expression will require further study.

### Supplementary Information

Supplementary Material 1. Figure S1. Perivascular structures. Figure S2. Schematic representation of cell culture in vitro. Figure S3. Schematic design and the establishment of conditional knock-in mice. Figure S4. EGLN3^CKI/CKI, Cdh5−creERT2^mice and PKM2^CKI/CKI, Aldh1|1−creERT2^ genotype identification. Figure S5. Validation of Shikonin inhibition of PKM2 expression. Figure S6. Verification of plasmid transfection. Table S1. PCR Primer Design Sequence.

Supplementary Material 2. Datasets for Molecular Docking.

## Data Availability

All data generated or analyzed during this study are included in this published article and supplemental materials. The datasets used and/or analyzed during the current study are available from the corresponding author on reasonable request.

## Abbreviations

SAH: Subarachnoid hemorrhage
BBB: Blood‒brain barrier
GLs: Glia limitans
TEER: Transverse electrical resistance
