# Supplementary material for: Endothelial EGLN3-PKM2 signaling induces the formation of acute astrocytic barrier to alleviate immune cell infiltration after subarachnoid hemorrhage
Source: Fluids Barriers CNS. 2024 May 16;21:42. doi: 10.1186/s12987-024-00550-8 (PMC11100217; doi:10.1186/s12987-024-00550-8)
Supplement: Supplementary file 1 — Supplementary Material 1. Figure S1. Perivascular structures. Figure S2. Schematic representation of cell culture in vitro. Figure S3. Schematic design and the establishment of conditional knock-in mice. Figure S4. EGLN3CKI/CKI, Cdh5−creERT2mice and PKM2CKI/CKI, Aldh1|1−creERT2 genotype identification. Figure S5. Validation of Shikonin inhibition of PKM2 expression. Figure S6. Verification of plasmid transfection. Table S1. PCR Primer Design Sequence. [file 12987_2024_550_MOESM1_ESM.docx]

**
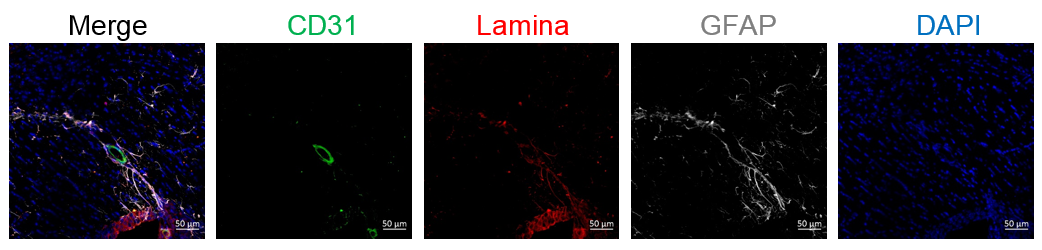
Supplementary Materials**

Fig. S1 Perivascular structures.

Immunofluorescent labeling of CD31(red) , Lamina-alpha(purple) , GFAP(green) ,and DAPI (blue) in the sham groups of C57BL/6 mice (scale bar, 50 μm). The structure of vascular and perivascular were vascular endothelial cells (and endothelial cell basement membrane), astrocyte basement membrane, astrocyte from inside to outside.


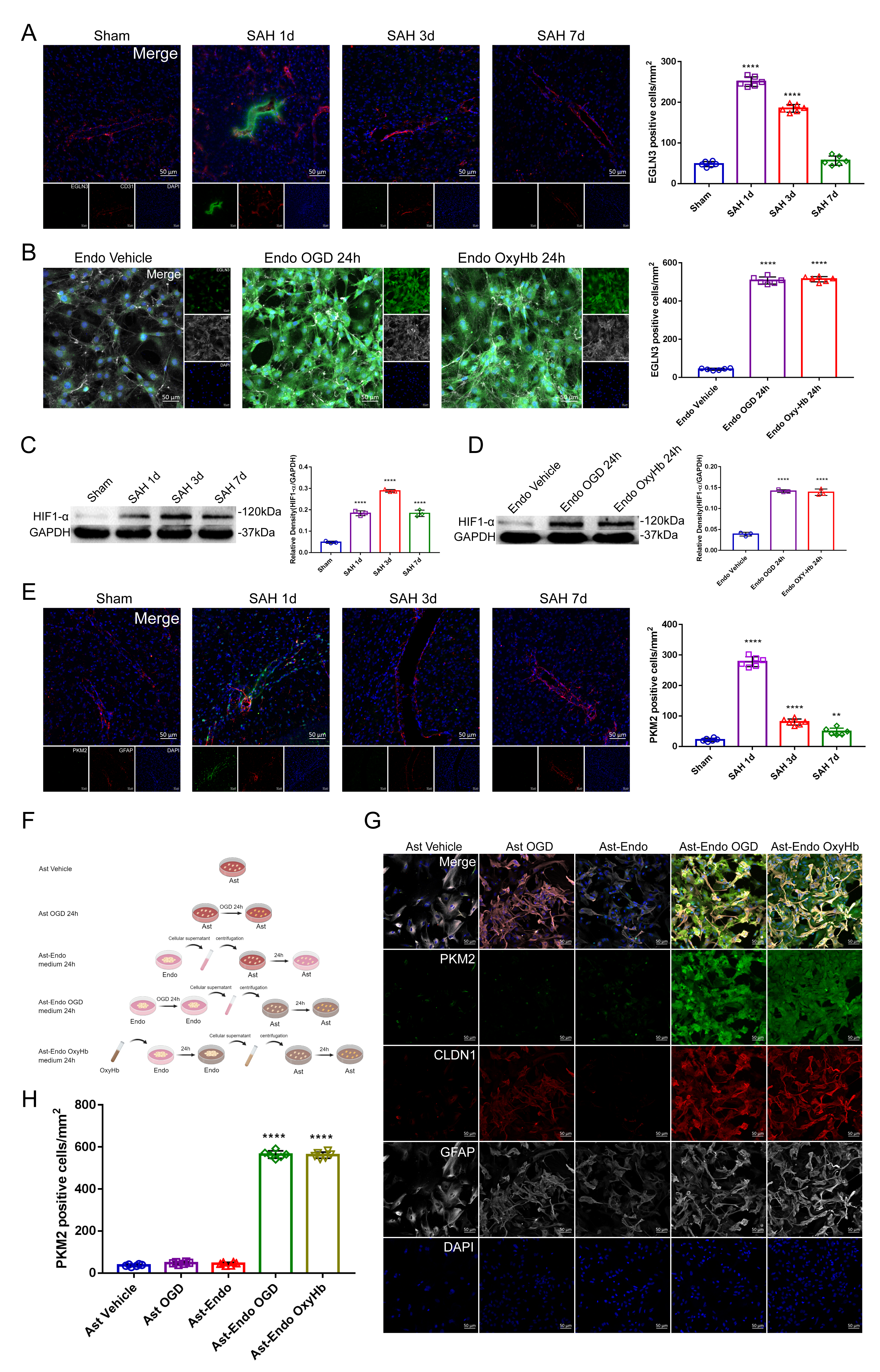


Fig. S2 Schematic representation of cell culture in vitro.

In the astrocyte vehicle group, astrocytes were cultured in astrocyte medium; in the astrocyte OGD group, astrocytes were treated for 24 h; in the ast-endo medium group, endothelial cells were cultured in endothelial cell medium for 24 h, and the cell supernatant was centrifuged and added to astrocytes that had already been removed from the supernatant; in the ast-endo OGD medium group, endothelial cells were treated 24 h after OGD, and the cell supernatant was centrifuged and added to astrocytes that had already been removed from the supernatant; and in the ast-endo OxyHb medium group, endothelial cells were cultured in endothelial cell medium supplemented with oxygen and hemoglobin for 24 h, and then the cell supernatant was centrifuged and added to astrocytes that had already been removed from the supernatant. Astrocytes from different groups were cultured for 24 h after treatment.


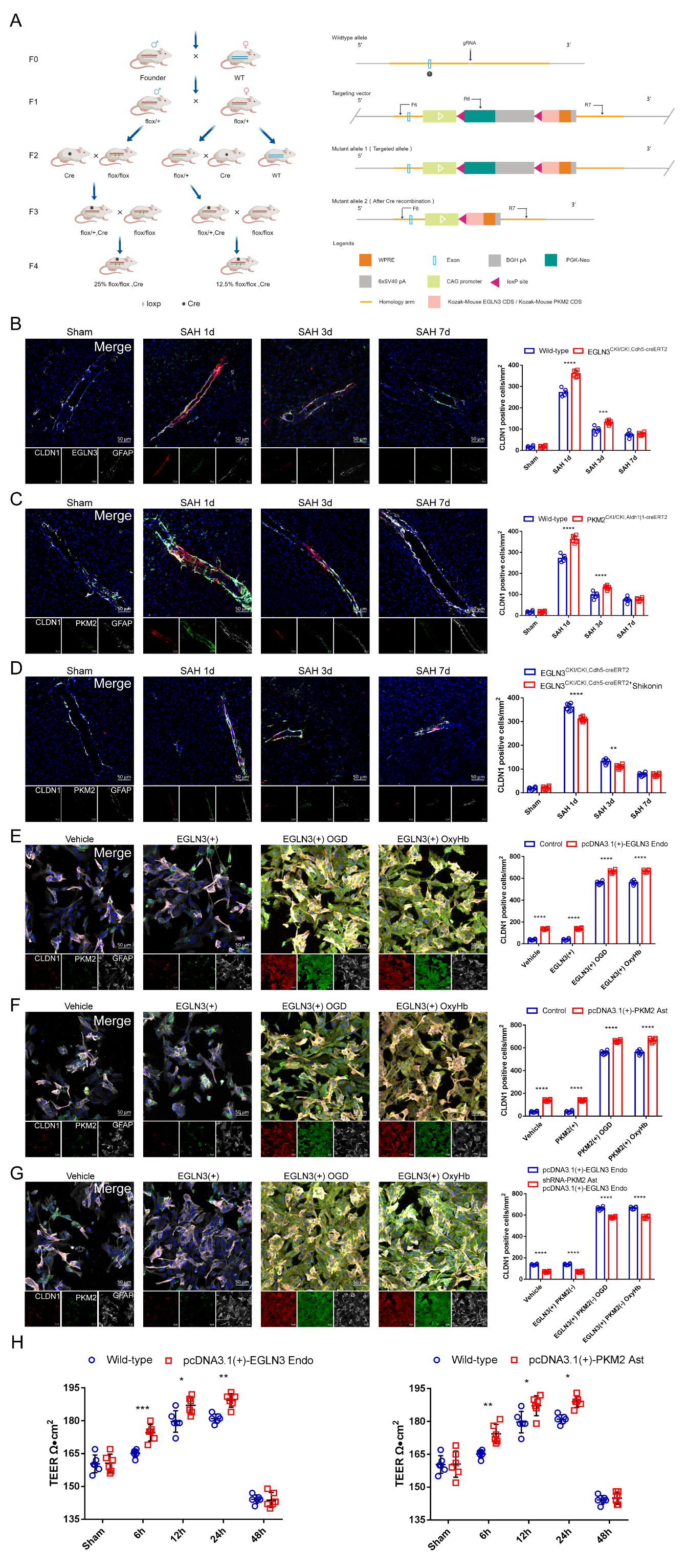


Fig. S3 Schematic design and the establishment of conditional knock-in mice.

Insertion of overexpressed EGLN3/PKM2 sequence with a stopper into the gene sequence by gRNA, and conditional overexpression of the target gene by conditionally knocking out the stopper.


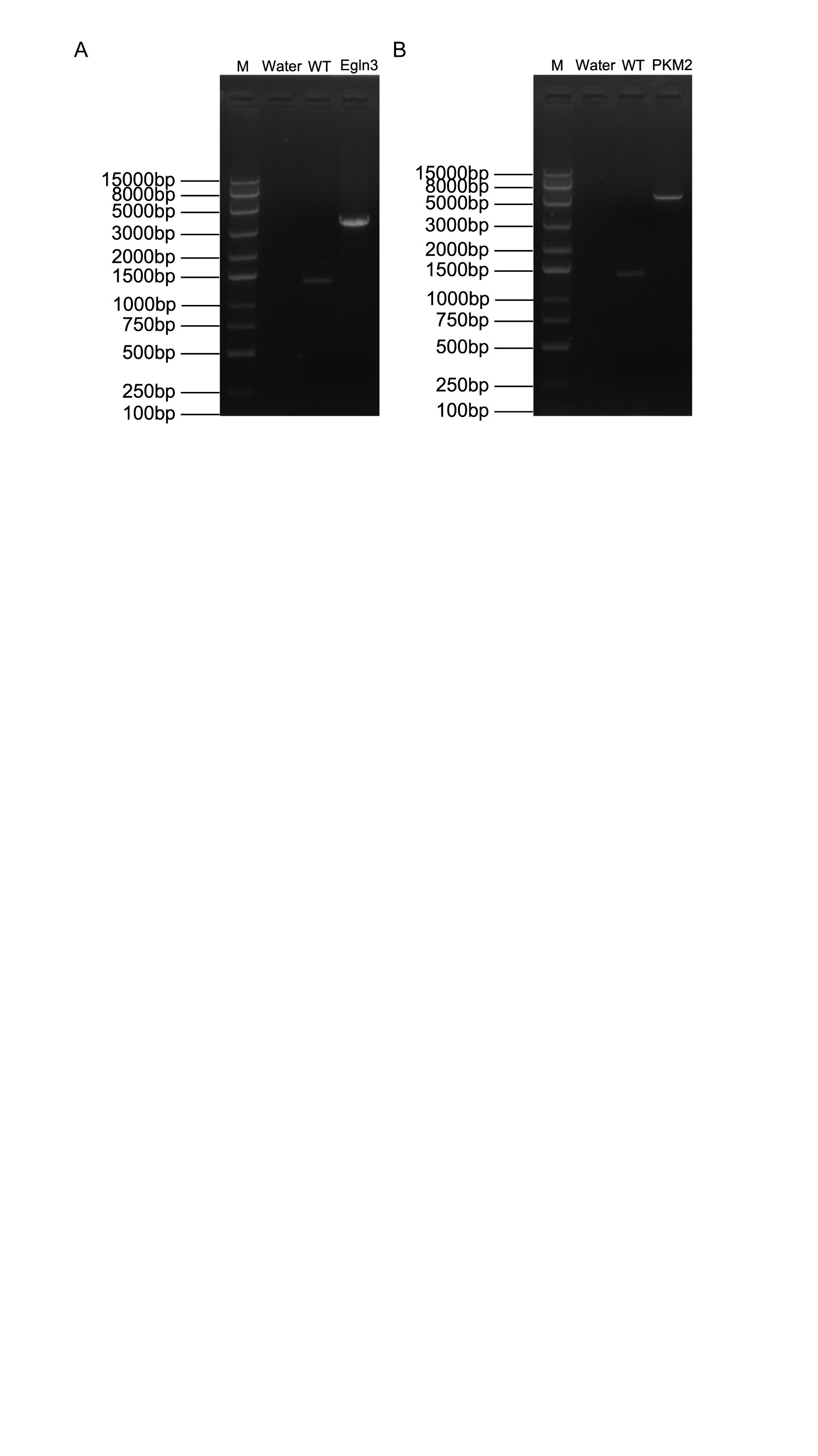


Fig. S4 EGLN3^CKI/CKI,Cdh5-creERT2^mice and PKM2^CKI/CKI,Aldh1| 1-creERT2^ genotype identification.

PCR results showed that EGLN3(A) and PKM2(B) genes had been successfully conditional knock-in transgenic mice. The length of CDS region of Egln3 and PKM2 genes were 729bp and 1596 bp, respectively. Therefore, the band length of wild-type mice was (F6 to R7) 1343bp; The length of the EGLN3^CKI/CKI^ mouse band is (F6 to R6) 3277bp and (F6 to R7) 6804bp; The length of the PKM2^CKI/CKI^ mouse band is (F6 to R6) 3277bp and (F6 to R7) 7680 bp; The length of the EGLN3^CKI/CKI,Cdh5-creERT2^ mouse band is (F6 to R7) 4643bp, PKM2^CKI/CKI,Aldh1| 1-creERT2^miceThe length of the band (F6 to R7) was 5519 bp. Primer sequences are shown in Table 2.


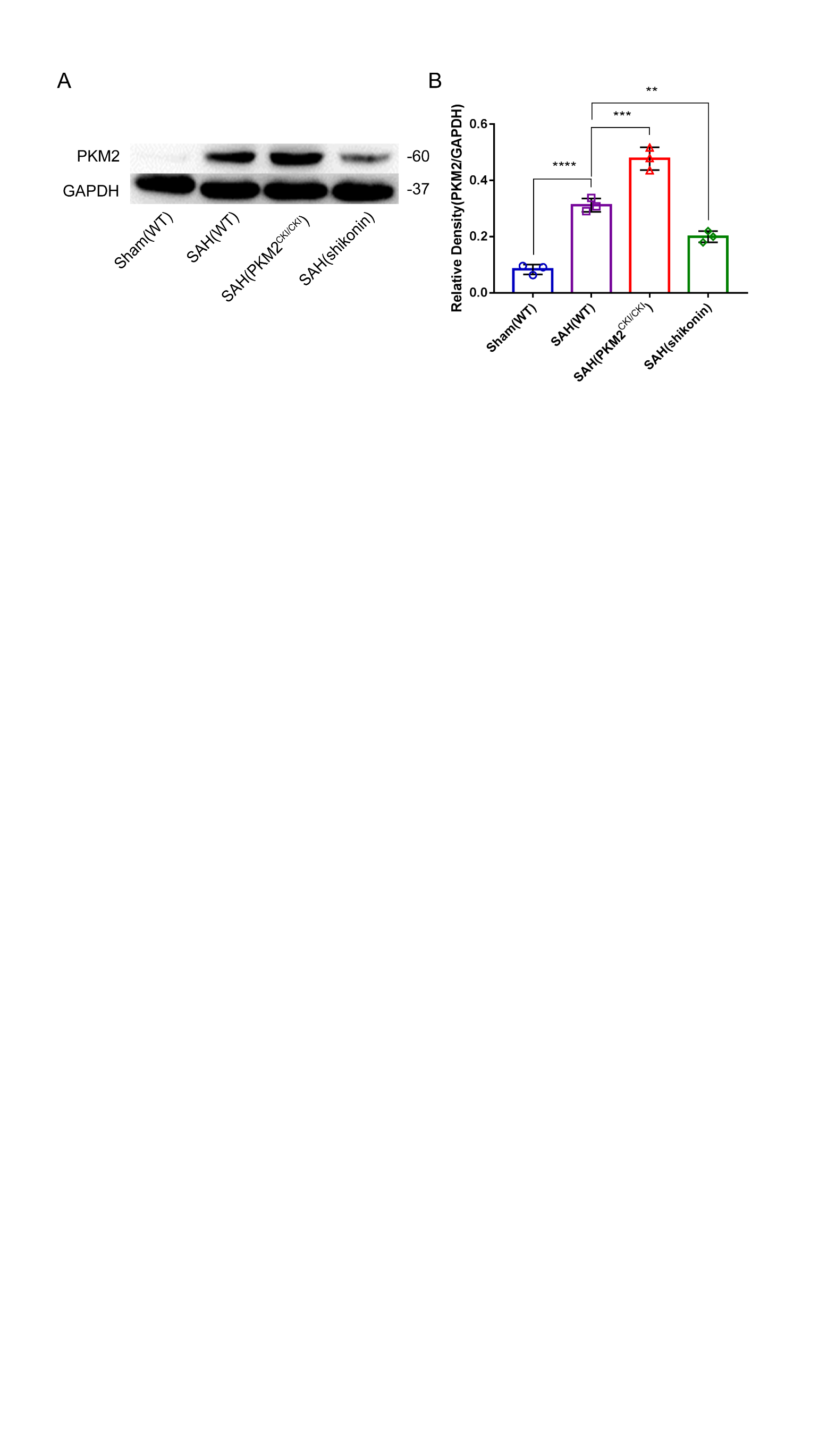


Fig. S5 Validation of shikonin inhibition of PKM2 expression.

Western blots experiment (A) and quantitative statistics (B) showed that PKM2 expression increased one day after SAH. The expression of PKM-2 was up-regulated in PKM2^CKI/CKI,Aldh1|1-creERT2^ mice compared with wild-type mice, and decreased in mice injected shikonin after SAH compared with wild-type mice. ** P < 0.01; *** P < 0.001; **** P < 0.0001.


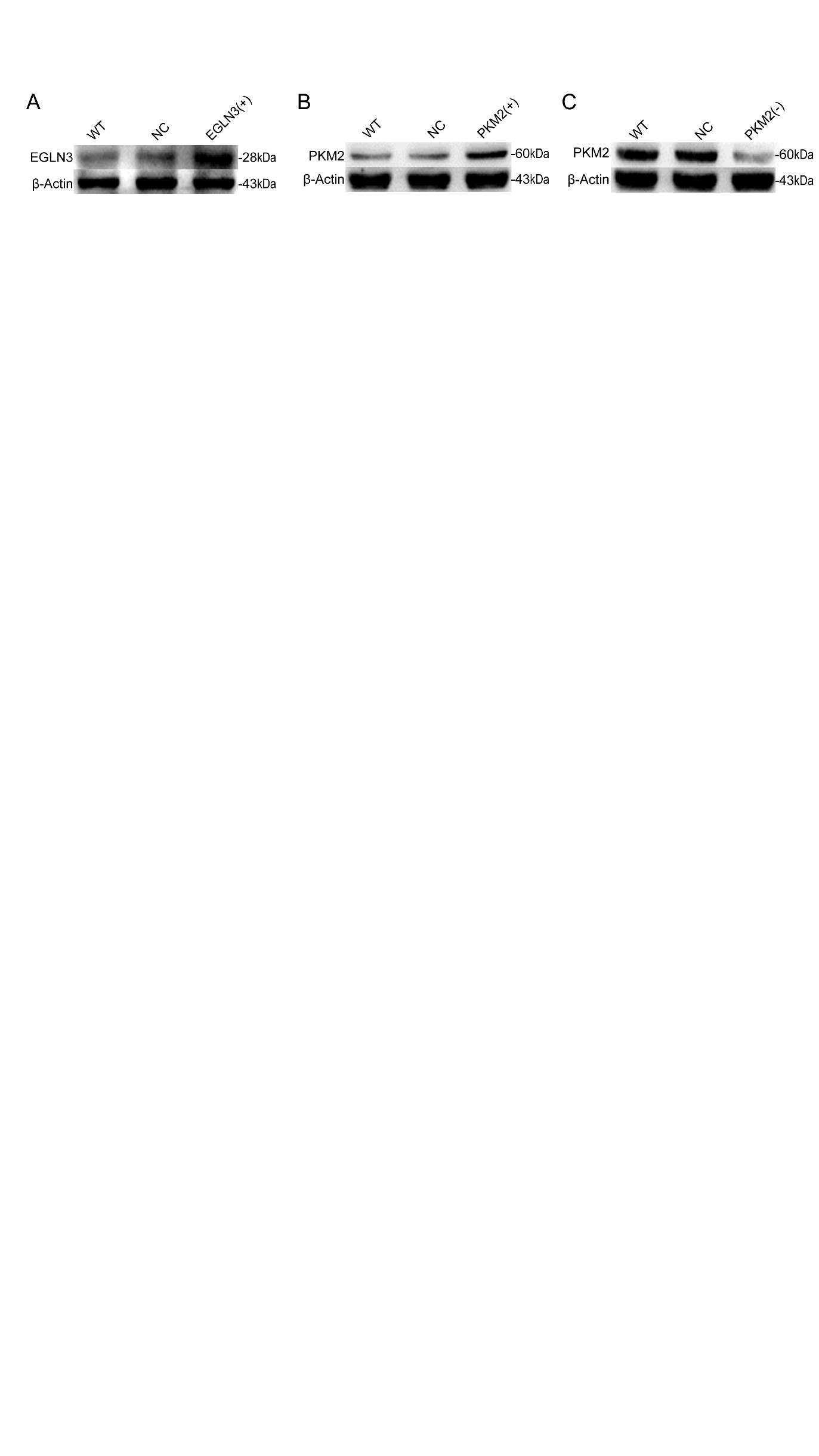


Fig. S6 Verification of plasmid transfection.

Western blots analysis showed that EGLN-3 was up-regulated in endothelial cells transfected with pcDNA3.1(+)-EGLN-3 (A), PKM-2 was up-regulated in astrocytes transfected with pcDNA3.1(+)-PKM-2 (B), and PKM-2 was down-regulated in astrocytes transfected with Psd1211-U6-shRNA-PKM2 (C).

Table S1 PCR Primer Design Sequence.

| **Target gene** | **Forward 6 (5'-3')** | **Reverse 6 (5'-3')** | **Reverse 7 (5'-3')** |
| --- | --- | --- | --- |
| EGLN3&PKM2 | AGGGAGGGTCAGCGAAAG | GCAGGAGCAAGGTGAGATG | GACGGGAGAGGTGATAGACAC |

EGLN3 and PKM2 conditional gene knock-in sites are located on chromosome 6 Rosa26 locus, so the primer sequence design is the same.

**Supplemental Methods**

The detail of the animals used was shown as follow:

**Experiment 1: Spatial changes and the localization of BBB and GL structures after SAH.**

| Tests | Sham  (WT) | SAH 1d  (WT) | SAH 3d  (WT) | SAH 7d  (WT) |
| --- | --- | --- | --- | --- |
| IF | 12 | 12 | 12 | 12 |
| TEM | 3 | 3 | 3 | 3 |

(Notes: Number of mice; Primary cells were selected from neonatal mice less than 3 days old, 3 mice per group, 9 mice in total ;Total number: 69)

**Experiment 2: GL barrier function and the effect of neurological outputs after SAH.**

| Tests | Sham  (WT) | SAH 1d  (WT) | SAH 3d  (WT) | SAH 7d  (WT) |
| --- | --- | --- | --- | --- |
| IF | 12 | 12 | 12 | 12 |
| EB absolute quantification | 6 | 6 | 6 | 6 |
| FCM | 6 | 6 | 6 | 6 |
| Open field test | 8 | 8 | 8 | 8 |
| Modified Garcia score | 8 | 8 | 8 | 8 |
| Beam balance score | 8 | 8 | 8 | 8 |
| Brain water content | 7 | 7 | 7 | 7 |

(Notes: Number of mice; Total number: 156)

**Experiment 3: Changes in the expression of EGLN3 and HIF-1 in endothelial cells after SAH.**

| Tests | Sham  (WT) | SAH 1d  (WT) | SAH 3d  (WT) | SAH 7d  (WT) |
| --- | --- | --- | --- | --- |
| IF | 6 | 6 | 6 | 6 |
| WB | 3 | 3 | 3 | 3 |

(Notes: Number of mice; Primary cells were selected from neonatal mice less than 3 days old, 3 mice per group, 9 mice in total; Total number: 45)

**Experiment 4: Changes in the expression of PKM2and CLDN1 in astrocytes after SAH.**

| Tests | Sham  (WT) | SAH 1d  (WT) | SAH 3d  (WT) | SAH 7d  (WT) |
| --- | --- | --- | --- | --- |
| IF | 6 | 6 | 6 | 6 |

(Notes: Number of mice; Primary cells were selected from neonatal mice less than 3 days old, 3 mice per group, 15 mice in total; Total number: 39)

**Experiment 5: Changes in the expression of EGLN3 and CLDN1 in astrocytes after SAH.**

| Tests | Sham  (WT) | SAH 1d  (WT) | SAH 3d  (WT) | SAH 7d  (WT) |
| --- | --- | --- | --- | --- |
| WB | 3 | 3 | 3 | 3 |
| CO-IP | 3 | 3 | 3 | 3 |

(Notes: Number of mice; Primary cells were selected from neonatal mice less than 3 days old, 3 mice per group, 9 mice in total; Total number: 33)

**Experiment 6: EGLN3 or PKM2 could enhance the tight junction expression of GL.**

| Animal genotype: EGLN3^CKI/CKI^ / PKM2^CKI/CKI^ / Shikonin-EGLN3^CKI/CKI^ | | | | |
| --- | --- | --- | --- | --- |
| Tests | Sham | SAH 1d | SAH 3d | SAH 7d |
| IF | 18 | 18 | 18 | 18 |

(Notes: Number of mice; Primary cells were selected from neonatal mice less than 3 days old, 3 mice per group, 36 mice in total; Total number: 108)

**Experiment 7: EGLN3 or PKM2 enhances barrier function of BBB.**

| Animal genotype: EGLN3^CKI/CKI^ / PKM2^CKI/CKI^ / Shikonin-EGLN3^CKI/CKI^ | | | | |
| --- | --- | --- | --- | --- |
| Tests | Sham | SAH 1d | SAH 3d | SAH 7d |
| IF | 12 | 12 | 12 | 12 |
| EB absolute quantification | 6 | 6 | 6 | 6 |
| FCM | 6 | 6 | 6 | 6 |

(Notes: Number of mice; Total number: 96)

**Experiment 8: EGLN3 and PKM2 Protect Neurological Function and Reduce Cerebral Edema.**

| Animal genotype: EGLN3^CKI/CKI^ / PKM2^CKI/CKI^ / Shikonin-EGLN3^CKI/CKI^ | | | | |
| --- | --- | --- | --- | --- |
| Tests | Sham | SAH 1d | SAH 3d | SAH 7d |
| Open field test | 8 | 8 | 8 | 8 |
| Modified Garcia score | 8 | 8 | 8 | 8 |
| Beam balance score | 8 | 8 | 8 | 8 |
| Brain water content | 8 | 8 | 8 | 8 |

(Notes: Number of mice; Total number: 64)

**Experiment 9: Expression of proteins downstream of PKM2 after SAH.**

| Tests | Sham | SAH 1d | SAH 3d | SAH 7d |
| --- | --- | --- | --- | --- |
| WB | 6 | 6 | 6 | 6 |

(Notes: Number of mice; Total number: 24)
